# Supplementary material for: Proximity-dependent mapping of the HCMV US28 interactome identifies RhoGEF signaling as a requirement for efficient viral reactivation
Source: PLoS Pathog. 2023 Oct 2;19(10):e1011682. doi: 10.1371/journal.ppat.1011682 (PMC10569644; doi:10.1371/journal.ppat.1011682)
Supplement: S1 Table — (PDF) [file ppat.1011682.s004.pdf]

**Supplemental Information Table S1. Pathway Analysis of US28 BirA Interactome in 293 Cells**

| Pathway Name | #Entities Total | #Entities Found | Entities pValue | Mapped Entities |
|---|---|---|---|---|
| Metabolism of proteins | 511 | 1983 | 2.99E-24 | [illegible] |
| Post-translational protein modification | 320 | 1392 | 2.48E-07 | [illegible] |
| Vesicle-mediated transport | 283 | 725 | 1.52E-44 | [illegible] |
| Membrane Trafficking | 278 | 626 | 3.21E-57 | [illegible] |
| Disease | 273 | 1040 | 2.49E-12 | [illegible] |
| Metabolism of RNA | 238 | 663 | 7.72E-30 | [illegible] |
| Signaling by Rho GTPases | 215 | 418 | 6.44E-06 | [illegible] |
| Cell Cycle | 190 | 602 | 3.24E-16 | [illegible] |
| Cellular responses to external stimuli | 153 | 473 | 7.91E-14 | [illegible] |
| Infectious disease | 141 | 372 | 3.28E-19 | [illegible] |
| M Phase | 133 | 354 | 1.03E-17 | [illegible] |
| Cellular responses to stress | 125 | 396 | 2.69E-10 | [illegible] |
| Translation | 119 | 294 | 1.22E-18 | [illegible] |
| Signaling by Receptor Tyrosine Kinases | 117 | 443 | 3.90E-05 | [illegible] |
| Neutrophil degranulation | 116 | 480 | 0.002878156 | [illegible] |
| HIV Infection | 107 | 229 | 2.31E-22 | [illegible] |
| Diseases of signal transduction | 106 | 373 | 3.81E-06 | [illegible] |
| Asparagine N-linked glycosylation | 105 | 304 | 4.77E-11 | [illegible] |
| Intra-Golgi and retrograde Golgi-to-ER traffic | 103 | 204 | 1.01E-24 | [illegible] |
| Processing of Capped Intron-Containing Pre-mRNA | 103 | 238 | 1.80E-18 | [illegible] |
| Transcriptional Regulation by TP53 | 97 | 362 | 0.000173175 | [illegible] |
| Intracellular signaling by second messengers | 91 | 287 | 1.84E-07 | [illegible] |
| RHO GTPase Effectors | 91 | 288 | 2.22E-07 | [illegible] |
| Cell Cycle Checkpoints | 89 | 272 | 5.55E-08 | [illegible] |
| Signaling by WNT | 81 | 297 | 0.000568035 | [illegible] |
| mRNA Splicing - Major Pathway | 80 | 180 | 5.70E-15 | [illegible] |
| mRNA Splicing | 80 | 188 | 1.11E-13 | [illegible] |
| PIP3 activates AKT signaling | 80 | 248 | 6.82E-07 | [illegible] |
| Deubiquitination | 80 | 282 | 0.00013879 | [illegible] |
| Organelle biogenesis and maintenance | 78 | 293 | 0.001924529 | [illegible] |
| Signaling by ROBO receptors | 76 | 217 | 3.58E-08 | [illegible] |
| Mitotic Anaphase | 75 | 195 | 3.49E-10 | [illegible] |
| Mitotic Metaphase and Anaphase | 75 | 196 | 4.59E-10 | [illegible] |
| ER to Golgi Anterograde Transport | 71 | 154 | 3.63E-14 | [illegible] |
| Transport to the Golgi and subsequent modification | 71 | 185 | 1.39E-09 | [illegible] |
| Host Interactions of HIV factors | 70 | 126 | 2.24E-19 | [illegible] |
| Separation of Sister Chromatids | 70 | 187 | 6.88E-09 | [illegible] |
| G2/M Transition | 70 | 196 | 7.05E-08 | [illegible] |
| Mitotic G2-G2/M phases | 70 | 198 | 1.12E-07 | [illegible] |
| MAPK family signaling cascades | 70 | 278 | 0.024664306 | [illegible] |
| Golgi-to-ER retrograde transport | 68 | 135 | 6.06E-16 | [illegible] |
| HIV Life Cycle | 67 | 149 | 1.17E-12 | [illegible] |
| Mitotic Prometaphase | 67 | 195 | 7.00E-07 | [illegible] |
| Apoptosis | 62 | 172 | 3.79E-07 | [illegible] |
| Programmed Cell Death | 62 | 175 | 7.00E-07 | [illegible] |
| Ub-specific processing proteases | 62 | 205 | 0.000204609 | [illegible] |
| Late Phase of HIV Life Cycle | 61 | 136 | 2.24E-11 | [illegible] |
| Signaling by NOTCH | 60 | 201 | 0.000458617 | [illegible] |
| Phospholipid metabolism | 59 | 212 | 0.004533644 | [illegible] |
| Clathrin-mediated endocytosis | 58 | 144 | 1.23E-08 | [illegible] |
| Regulation of expression of SLITs and ROBOs | 57 | 171 | 2.05E-05 | [illegible] |
| TCF dependent signaling in response to WNT | 57 | 202 | 0.003966057 | [illegible] |
| S Phase | 55 | 161 | 1.39E-05 | [illegible] |
| PTEN Regulation | 54 | 138 | 1.68E-07 | [illegible] |
| [illegible] | 54 | 173 | 0.000319256 | [illegible] |
| Rab regulation of trafficking | 53 | 121 | 2.10E-09 | [illegible] |
| G2/M Checkpoints | 53 | 151 | 9.42E-06 | [illegible] |
| Signaling by Hedgehog | 52 | 150 | 1.76E-05 | [illegible] |
| Interleukin-1 family signaling | 50 | 137 | 5.81E-06 | [illegible] |
| COPI-mediated anterograde transport | 48 | 102 | 9.13E-10 | [illegible] |
| DNA Replication | 48 | 127 | 3.21E-06 | [illegible] |
| Mitochondrial translation elongation | 47 | 88 | 5.80E-12 | [illegible] |
| Mitochondrial translation | 47 | 94 | 1.29E-10 | [illegible] |
| COPI-dependent Golgi-to-ER retrograde traffic | 47 | 101 | 2.39E-09 | [illegible] |
| Synthesis of DNA | 47 | 119 | 1.02E-06 | [illegible] |
| Beta-catenin independent WNT signaling | 47 | 143 | 0.000285868 | [illegible] |
| Mitotic G1-G1/S phases | 47 | 148 | 0.000761144 | [illegible] |
| Clathrin derived vesicle budding | 46 | 72 | 9.22E-16 | [illegible] |
| trans-Golgi Network Vesicle Budding | 46 | 72 | 9.22E-16 | [illegible] |
| Mitotic Prophase | 46 | 104 | 3.08E-08 | [illegible] |
| G1/S Transition | 46 | 131 | 5.22E-05 | [illegible] |
| Influenza Infection | 46 | 153 | 0.003927022 | [illegible] |
| Mitochondrial translation initiation | 45 | 88 | 1.39E-10 | [illegible] |
| Mitochondrial translation termination | 45 | 88 | 1.39E-10 | [illegible] |
| [illegible] | 45 | 158 | 0.016733982 | [illegible] |
| [illegible] | 44 | 86 | 2.42E-10 | [illegible] |
| Cellular response to heat stress | 44 | 88 | 5.91E-10 | [illegible] |
| RHO GTPases Activate Formins | 44 | 136 | 0.000845418 | [illegible] |
| C-type lectin receptors (CLRs) | 44 | 140 | 0.001842331 | [illegible] |
| Resolution of Sister Chromatid Cohesion | 43 | 123 | 0.000131559 | [illegible] |
| Influenza Life Cycle | 43 | 142 | 0.005561123 | [illegible] |
| Disorders of transmembrane transporters | 43 | 144 | 0.007783453 | [illegible] |
| Interleukin-1 signaling | 42 | 101 | 1.08E-06 | [illegible] |
| ABC-family proteins mediated transport | 42 | 103 | 2.03E-06 | [illegible] |
| Hedgehog 'off' state | 42 | 114 | 3.81E-05 | [illegible] |
| Transcriptional regulation by RUNX2 | 41 | 118 | 0.000252719 | [illegible] |
| TCR signaling | 41 | 136 | 0.008826908 | [illegible] |
| MHC class II antigen presentation | 41 | 138 | 0.01207591 | [illegible] |
| Influenza Viral RNA Transcription and Replication | 40 | 133 | 0.010938977 | [illegible] |
| Golgi Associated Vesicle Biogenesis | 39 | 54 | 6.29E-16 | [illegible] |
| CLEC7A (Dectin-1) signaling | 39 | 97 | 8.63E-06 | [illegible] |
| Cargo recognition for clathrin-mediated endocytosis | 39 | 104 | 5.66E-05 | [illegible] |
| [illegible] | 39 | 123 | 0.003966057 | [illegible] |
| Regulation of HSF1-mediated heat shock response | 38 | 68 | 2.49E-10 | [illegible] |
| RAB GEFs exchange GTP for GDP on RABs | 38 | 89 | 2.19E-06 | [illegible] |
| Switching of origins to a post-replicative state | 38 | 90 | 3.11E-06 | [illegible] |
| tRNA processing | 38 | 104 | 0.000154934 | [illegible] |
| Transport of Mature Transcript to Cytoplasm | 37 | 80 | 3.41E-07 | [illegible] |
| PCP/CE pathway | 37 | 92 | 1.66E-05 | [illegible] |
| Antiviral mechanism by IFN-stimulated genes | 36 | 79 | 7.50E-07 | [illegible] |
| [illegible] | 36 | 82 | 2.19E-06 | [illegible] |
| DNA Replication Pre-Initiation | 36 | 85 | 6.03E-06 | [illegible] |
| APC/C-mediated degradation of cell cycle proteins | 36 | 86 | 8.24E-06 | [illegible] |
| Regulation of mitotic cell cycle | 36 | 86 | 8.24E-06 | [illegible] |
| MAPK6/MAPK4 signaling | 36 | 89 | 2.02E-05 | [illegible] |
| Unfolded Protein Response (UPR) | 36 | 94 | 8.39E-05 | [illegible] |
| Gene Silencing by RNA | 36 | 103 | 0.000795643 | [illegible] |
| Mitotic Spindle Checkpoint | 36 | 108 | 0.002423456 | [illegible] |
| ISG15 antiviral mechanism | 35 | 71 | 1.28E-07 | [illegible] |
| [illegible] | 35 | 80 | 3.64E-06 | [illegible] |
| Downstream signaling events of B Cell Receptor (BCR) | 35 | 81 | 5.21E-06 | [illegible] |
| Signaling by VEGF | 35 | 104 | 0.002465313 | [illegible] |
| Orc1 removal from chromatin | 34 | 71 | 4.77E-07 | [illegible] |
| [illegible] | 34 | 71 | 4.77E-07 | [illegible] |
| [illegible] | 34 | 72 | 6.96E-07 | [illegible] |
| VEGFA-VEGFR2 Pathway | 34 | 95 | 0.000788747 | [illegible] |
| Downstream TCR signaling | 34 | 115 | 0.041749932 | [illegible] |
| [illegible] | 34 | 116 | 0.049005663 | [illegible] |
| Nonsense-Mediated Decay (NMD) | 34 | 116 | 0.049005663 | [illegible] |
| COPII-mediated vesicle transport | 33 | 68 | 5.28E-07 | [illegible] |
| ABC transporter disorders | 33 | 76 | 9.71E-06 | [illegible] |
| [illegible] | 33 | 76 | 9.71E-06 | [illegible] |
| TP53 Regulates Metabolic Genes | 33 | 83 | 8.80E-05 | [illegible] |
| PI Metabolism | 33 | 84 | 0.000118558 | [illegible] |
| Hedgehog 'on' state | 33 | 85 | 0.000156708 | [illegible] |
| Regulation of PLK1 Activity at G2/M Transition | 33 | 85 | 0.000156708 | [illegible] |
| Respiratory electron transport | 33 | 100 | 0.006033184 | [illegible] |
| Retrograde transport at the Trans-Golgi-Network | 32 | 49 | 4.85E-11 | [illegible] |
| Defective CFTR causes cystic fibrosis | 32 | 61 | 9.80E-08 | [illegible] |
| Activation of NF-kappaB in B cells | 32 | 67 | 1.22E-06 | [illegible] |
| Regulation of RUNX2 expression and activity | 32 | 71 | 5.81E-06 | [illegible] |
| [illegible] | 32 | 72 | 8.24E-06 | [illegible] |
| [illegible] | 32 | 73 | 1.15E-05 | [illegible] |
| [illegible] | 32 | 75 | 2.21E-05 | [illegible] |
| [illegible] | 32 | 79 | 8.03E-05 | [illegible] |
| Signaling by NOTCH4 | 32 | 81 | 0.00014608 | [illegible] |
| Glucose metabolism | 32 | 86 | 0.000588227 | [illegible] |
| [illegible] | 32 | 92 | 0.002522921 | [illegible] |
| Amplification of signal from the kinetochores | 32 | 92 | 0.002522921 | [illegible] |
| [illegible] | 32 | 99 | 0.011346541 | [illegible] |
| Metabolism of non-coding RNA | 31 | 50 | 6.71E-10 | [illegible] |
| snRNP Assembly | 31 | 50 | 6.71E-10 | [illegible] |
| RAB geranylgeranylation | 31 | 65 | 2.07E-06 | [illegible] |
| Assembly of the pre-replicative complex | 31 | 68 | 6.65E-06 | [illegible] |
| Regulation of PTEN stability and activity | 31 | 69 | 9.45E-06 | [illegible] |
| Recruitment of NuMA to mitotic centrosomes | 31 | 94 | 0.009653083 | [illegible] |
| Anchoring of the basal body to the plasma membrane | 31 | 96 | 0.014178946 | [illegible] |
| UCH proteinases | 31 | 96 | 0.014178946 | [illegible] |
| Dectin-1 mediated noncanonical NF-kB signaling | 30 | 60 | 9.62E-07 | [illegible] |
| Degradation of GLI1 by the proteasome | 30 | 60 | 9.62E-07 | [illegible] |
| Degradation of GLI2 by the proteasome | 30 | 60 | 9.62E-07 | [illegible] |
| GLI3 is processed to GLI3R by the proteasome | 30 | 60 | 9.62E-07 | [illegible] |
| Macroautophagy | 30 | 68 | 2.20E-05 | [illegible] |
| Regulation of RAS by GAPs | 30 | 68 | 2.20E-05 | [illegible] |
| [illegible] | 30 | 72 | 8.57E-05 | [illegible] |
| Transcriptional regulation by small RNAs | 30 | 73 | 0.000118558 | [illegible] |
| Transcriptional regulation by RUNX3 | 30 | 95 | 0.026003772 | [illegible] |
| Deadenylation-dependent mRNA decay | 29 | 56 | 6.96E-07 | [illegible] |
| NIK-->noncanonical NF-kB signaling | 29 | 59 | 2.52E-06 | [illegible] |
| Hedgehog ligand biogenesis | 29 | 65 | 2.59E-05 | [illegible] |
| [illegible] | 29 | 66 | 3.67E-05 | [illegible] |
| Glycolysis | 29 | 67 | 5.17E-05 | [illegible] |
| Cellular response to hypoxia | 29 | 75 | 0.0006453 | [illegible] |
| Regulation of Hypoxia-inducible Factor (HIF) by oxygen | 29 | 75 | 0.0006453 | [illegible] |
| Negative regulation of NOTCH4 signaling | 28 | 53 | 6.96E-07 | [illegible] |
| Vif-mediated degradation of APOBEC3G | 28 | 53 | 6.96E-07 | [illegible] |
| [illegible] | 28 | 55 | 1.75E-06 | [illegible] |
| Degradation of DVL | 28 | 57 | 4.25E-06 | [illegible] |
| Autodegradation of Cdh1 by Cdh1:APC/C | 28 | 63 | 4.24E-05 | [illegible] |
| APC/C:Cdc20 mediated degradation of Securin | 28 | 67 | 0.000169678 | [illegible] |
| AURKA Activation by TPX2 | 28 | 71 | 0.000605371 | [illegible] |
| CDK-mediated phosphorylation and removal of Cdc6 | 28 | 72 | 0.000795643 | [illegible] |
| Centrosome maturation | 28 | 80 | 0.006439074 | [illegible] |
| [illegible] | 28 | 80 | 0.006439074 | [illegible] |
| Cyclin E associated events during G1/S transition | 28 | 83 | 0.012575129 | [illegible] |
| Metabolism of polyamines | 28 | 84 | 0.015580279 | [illegible] |
| Cyclin A:Cdk2-associated events at S phase entry | 28 | 85 | 0.019048798 | [illegible] |
| Nuclear Envelope Breakdown | 27 | 47 | 1.50E-07 | [illegible] |
| tRNA processing in the nucleus | 27 | 55 | 7.14E-06 | [illegible] |
| [illegible] | 27 | 56 | 1.06E-05 | [illegible] |
| CDT1 association with the CDC6:ORC:origin complex | 27 | 59 | 3.44E-05 | [illegible] |
| Hh mutants abrogate ligand secretion | 27 | 59 | 3.44E-05 | [illegible] |
| Asymmetric localization of PCP proteins | 27 | 64 | 0.000202448 | [illegible] |
| COPI-independent Golgi-to-ER retrograde traffic | 26 | 53 | 1.17E-05 | [illegible] |
| AUF1 (hnRNP D0) binds and destabilizes mRNA | 26 | 54 | 1.76E-05 | [illegible] |
| Regulation of RUNX3 expression and activity | 26 | 55 | 2.65E-05 | [illegible] |
| SCF-beta-TrCP mediated degradation of Emi1 | 26 | 55 | 2.65E-05 | [illegible] |
| SCF(Skp2)-mediated degradation of p27/p21 | 26 | 60 | 0.000173035 | [illegible] |
| Loss of Nlp from mitotic centrosomes | 26 | 68 | 0.002189612 | [illegible] |
| [illegible] | 26 | 68 | 0.002189612 | [illegible] |
| Signaling by MET | 26 | 77 | 0.019956459 | [illegible] |
| [illegible] | 25 | 50 | 1.26E-05 | [illegible] |
| [illegible] | 25 | 50 | 1.26E-05 | [illegible] |
| Ubiquitin-dependent degradation of Cyclin D | 25 | 52 | 2.95E-05 | [illegible] |
| Ubiquitin-dependent degradation of Cyclin D1 | 25 | 52 | 2.95E-05 | [illegible] |
| Vpu mediated degradation of CD4 | 25 | 52 | 2.95E-05 | [illegible] |
| Regulation of Apoptosis | 25 | 53 | 4.41E-05 | [illegible] |
| Degradation of AXIN | 25 | 55 | 0.000100019 | [illegible] |
| Interactions of Rev with host cellular proteins | 24 | 34 | 3.28E-09 | [illegible] |
| [illegible] | 24 | 39 | 1.84E-07 | [illegible] |
| [illegible] | 24 | 40 | 3.49E-07 | [illegible] |
| TBC/RABGAPs | 24 | 44 | 3.18E-06 | [illegible] |
| Regulation of ornithine decarboxylase (ODC) | 24 | 51 | 7.75E-05 | [illegible] |
| Autodegradation of the E3 ubiquitin ligase COP1 | 24 | 52 | 0.000116418 | [illegible] |
| p53-Independent DNA Damage Response | 24 | 53 | 0.000167342 | [illegible] |
| p53-Independent G1/S DNA damage checkpoint | 24 | 53 | 0.000167342 | [illegible] |
| [illegible] | 24 | 53 | 0.000167342 | [illegible] |
| Stabilization of p53 | 24 | 57 | 0.000711869 | [illegible] |
| p53-Dependent G1 DNA Damage Response | 24 | 66 | 0.010020009 | [illegible] |
| p53-Dependent G1/S DNA damage checkpoint | 24 | 66 | 0.010020009 | [illegible] |
| G1/S DNA Damage Checkpoints | 24 | 68 | 0.016156615 | [illegible] |
| [illegible] | 24 | 72 | 0.039771542 | [illegible] |
| Rev-mediated nuclear export of HIV RNA | 23 | 32 | 4.84E-09 | [illegible] |
| Interactions of Vpr with host cellular proteins | 23 | 34 | 3.12E-08 | [illegible] |
| NS1 Mediated Effects on Host Pathways | 23 | 38 | 5.51E-07 | [illegible] |
| Host Interactions with Influenza Factors | 23 | 40 | 1.75E-06 | [illegible] |
| Viral Messenger RNA Synthesis | 23 | 41 | 3.11E-06 | [illegible] |
| tRNA Aminoacylation | 23 | 42 | 5.40E-06 | [illegible] |
| mRNA Splicing - Minor Pathway | 23 | 52 | 0.000423279 | [illegible] |
| Synthesis of PIPs at the plasma membrane | 23 | 53 | 0.000608273 | [illegible] |
| XBP1(S) activates chaperone genes | 23 | 54 | 0.000845865 | [illegible] |
| IRE1alpha activates chaperones | 23 | 56 | 0.00169739 | [illegible] |
| [illegible] | 23 | 57 | 0.002301322 | [illegible] |
| [illegible] | 23 | 66 | 0.025681418 | [illegible] |
| RNA Polymerase II Transcription Termination | 23 | 66 | 0.025681418 | [illegible] |
| Vpr-mediated nuclear import of PICs | 22 | 31 | 1.93E-08 | [illegible] |
| Nuclear Pore Complex (NPC) Disassembly | 22 | 33 | 1.12E-07 | [illegible] |
| mTOR signalling | 22 | 40 | 8.81E-06 | [illegible] |
| SUMOylation of DNA replication proteins | 22 | 43 | 3.80E-05 | [illegible] |
| SUMOylation of RNA binding proteins | 22 | 43 | 3.80E-05 | [illegible] |
| SUMOylation of chromatin organization proteins | 22 | 53 | 0.002052751 | [illegible] |
| Complex I biogenesis | 22 | 55 | 0.003874605 | [illegible] |
| Ribosomal scanning and start codon recognition | 22 | 59 | 0.01207591 | [illegible] |
| Translation initiation complex formation | 22 | 59 | 0.01207591 | [illegible] |
| [illegible] | 22 | 60 | 0.015647315 | [illegible] |
| Nuclear import of Rev protein | 21 | 31 | 1.68E-07 | [illegible] |
| Transport of the SLBP independent Mature mRNA | 21 | 32 | 3.56E-07 | [illegible] |
| Transport of the SLBP Dependant Mature mRNA | 21 | 33 | 6.98E-07 | [illegible] |
| Signaling by EGFR | 21 | 48 | 0.001229003 | [illegible] |
| NEP/NS2 Interacts with the Cellular Export Machinery | 20 | 29 | 2.47E-07 | [illegible] |
| Transport of Ribonucleoproteins into the Host Nucleus | 20 | 29 | 2.47E-07 | [illegible] |
| Export of Viral Ribonucleoproteins from Nucleus | 20 | 30 | 5.45E-07 | [illegible] |
| SUMOylation of ubiquitinylation proteins | 20 | 35 | 1.38E-05 | [illegible] |
| Intra-Golgi traffic | 20 | 44 | 0.000985892 | [illegible] |
| Energy dependent regulation of mTOR by LKB1-AMPK | 19 | 29 | 1.71E-06 | [illegible] |
| [illegible] | 19 | 29 | 1.71E-06 | [illegible] |
| SUMOylation of SUMOylation proteins | 19 | 32 | 1.26E-05 | [illegible] |
| [illegible] | 19 | 52 | 0.04033264 | [illegible] |
| Signaling by ERBB2 | 18 | 47 | 0.029402128 | [illegible] |
| Budding and maturation of HIV virion | 16 | 28 | 0.000195303 | [illegible] |
| [illegible] | 16 | 31 | 0.001004904 | [illegible] |
| Lysosome Vesicle Biogenesis | 16 | 35 | 0.006033184 | [illegible] |
| MAP2K and MAPK activation | 16 | 40 | 0.034115211 | [illegible] |
| Cytosolic tRNA aminoacylation | 14 | 24 | 0.000588227 | [illegible] |
