# Supplementary material for: Proximity-dependent mapping of the HCMV US28 interactome identifies RhoGEF signaling as a requirement for efficient viral reactivation
Source: PLoS Pathog. 2023 Oct 2;19(10):e1011682. doi: 10.1371/journal.ppat.1011682 (PMC10569644; doi:10.1371/journal.ppat.1011682)
Supplement: S2 Table — (PDF) [file ppat.1011682.s005.pdf]

Supplemental Information Table S2. Pathway Analysis of HCMV-US28 BirA Interactome in NHDFs

Pathway name	#Entities Found	#Entities Total	Entities pValue	Mapped entities
Vesicle-mediated transport	166	762	1.11E-16	Q14203;P53365;Q9BYX2;O15084;O75146;Q9H444;Q9P299;Q96L93;Q9UPN7;P61020;Q9Y6G9;Q92572;Q0JRZ9;P48444;Q92574;Q92734;O95721;Q9UIQ6;P49257;Q5JRA6;Q9UBC2;Q9P1Q0;Q8IWJ2;Q9Y4L1;P86790;O00161;Q96CN9;Q13492;P00533;Q15276;Q9Y6Y8;Q15836;P48553;Q96D71;Q14746;Q8WVM8;O75886;
Membrane Trafficking	164	636	1.11E-16	Q14203;P53365;Q9BYX2;O15084;O75146;Q9H444;Q9P299;Q96L93;Q9UPN7;P61020;Q9Y6G9;Q92572;Q0JRZ9;P48444;Q92574;Q92734;O95721;Q9UIQ6;P49257;Q5JRA6;Q9UBC2;Q9P1Q0;Q8IWJ2;P86790;O00161;Q96CN9;Q13492;P00533;Q15276;Q9Y6Y8;Q15836;P48553;Q96D71;Q14746;Q8WVM8;O75886;O95295;
Signaling by Rho GTPases	117	678	3.55E-07	Q2M1Z3;Q9Y266;Q9Y6W5;P12814;P12931;Q9P2R3;Q9Y6G9;Q9UH65;Q8IWB9;Q8WUM0;P27105;Q9NXC2;Q96HY6;A0JNW5;P49257;Q16512;Q16513;Q8IZP0;Q658Y4;Q14739;Q9H792;O60610;Q96RT1;O00161;Q9Y4D1;Q9HD26;Q9BZH6;Q5JTV8;P41240;Q86XL3;Q13492;Q14344;Q15311;P42336;Q9H2G2;A4D1P6;Q9U
Intra-Golgi and retrograde Golgi-to-ER traffic	56	206	5.68E-10	Q9UIL1;Q9UBF2;Q14203;P53365;Q92538;Q9H2M9;O43752;Q9NP61;Q9UID3;Q9P299;O43237;Q96L93;Q9Y6G9;Q9UJW0;Q8WTW3;P48444;Q15102;Q92805;O95721;P51151;Q9P1Q0;Q9P2W9;Q8IWJ2;Q4ADV7;O60499;Q96CN9;P46459;O43731;Q15836;Q14746;P33176;P84085;Q5VIR6;Q9NZ43;O60664;Q9H0B6;Q150
M Phase	54	393	0.038341582	P52948-3;P52948-4;Q92539;O95476;Q9Y266;Q14203-2;Q14008;P42167-1;Q9H444;Q9H8Y8;O43237;P51665;P35658;P52948-5;Q9Y6G9;Q99567;P61981;O60763;P62195;Q8WUM0;Q96HA1;Q14693;P46060;Q66LE6;Q86Y07-2;O75694;P30153;P36873;Q14739;P19784;Q8WUX9;Q9UQE7;Q86XL3;Q8WYP5;Q9H2G9;Q16401;Q9Y
Asparagine N-linked glycosylation	51	305	0.001426122	Q9UBF2;Q14203;Q92538;O15084;Q9NP61;Q9P299;O43237;Q12907;Q9UPN7;Q9NYU2;Q9Y6G9;O60763;Q9UJW0;Q8WTW3;P48444;Q92734;Q9NRC6;Q8IUR0;O95487;Q9Y673;P49257;Q5JRA6;O95486;Q9UJ70;Q9HCU5;P46459;Q9Y6Y8;O43731;P48553;Q14746;Q8WVM8;Q14789;P84085;Q9GZP9;O15498;P83436;Q9N
ER to Golgi Anterograde Transport	43	155	3.21E-08	Q9Y6Y8;Q14203;Q9UBF2;O15084;Q92538;O43731;Q9NP61;P48553;Q14746;Q14789;Q8WVM8;Q9P299;O43237;Q12907;Q9UPN7;P84085;Q9Y6G9;O60763;Q9UJW0;Q8WTW3;P48444;O15498;P83436;Q92734;Q9NRC6;Q8IUR0;Q12893;O95487;O94855;O15155;P49257;Q5JRA6;O75396;O95486;Q96FJ2;Q13190;O150
Transport to the Golgi and subsequent modification	43	186	3.60E-06	Q9Y6Y8;Q14203;Q9UBF2;O15084;Q92538;O43731;Q9NP61;P48553;Q14746;Q14789;Q8WVM8;Q9P299;O43237;Q12907;Q9UPN7;P84085;Q9Y6G9;O60763;Q9UJW0;Q8WTW3;P48444;O15498;P83436;Q92734;Q9NRC6;Q8IUR0;Q12893;O95487;O94855;O15155;P49257;Q5JRA6;O75396;O95486;Q96FJ2;Q13190;O150
Clathrin-mediated endocytosis	41	145	4.03E-08	P25098;Q14247;P52594;Q96D71;Q15836;O75886;O75146;Q2M2I8;Q8WXE9;Q9Y6I3;O00443;Q14C86;O00401;Q5SW96;P61020;P51809;P20339;O00291;Q99961;Q9Y5X1;Q0JRZ9;Q9NZM3;P98082;P61201;Q15642;O14964;Q01968;P42566;P11717;O94973;Q9BT88;O15056;Q92905;Q9UBC2;Q9NVZ3;O43426;Q13492;P
Phospholipid metabolism	36	211	0.004901243	Q92539;O95674;Q9NP80;P48651;Q13614;Q96QG7;Q99570;Q9Y2I7;Q8IV08;Q53EU6;O00443;Q86T03;P20339;Q96N66;Q01968;Q14693;Q9BTU6;Q8TBX8;O94830;Q92604;Q9Y217;O15056;Q08AM6;Q9NRZ7;O43426;Q86UL3;P19784;Q9H0X9;Q9Y2H2;Q9BZF1;Q9C0D9;Q96T51;Q13393;Q9HB20;P42336;O15327
RAC1 GTPase cycle	35	185	0.001088052	Q9Y2U8;Q2M1Z3;Q15836;Q9Y6W5;Q03135;Q5VT25;Q96HP0;O00401;Q9Y2A7;P42167;Q96N67;Q86Y07;Q9UH65;O15498;P05556;Q96BY6;Q16512;Q16513;Q6IAA8;Q8IZP0;Q5T5U3;Q14739;Q12979;Q9NSV4;Q07960;P50402;Q96RT1;O00161;Q13393;P43121;Q13177;P29317;Q14344;Q15311;P42336
Mitotic Anaphase	35	237	0.036969162	Q9Y2U8;Q9Y266;Q14008;P42167-1;Q9BTX1;Q9H444;Q9UQN3;O43237;P51665;P35998;P52948-5;Q9Y6G9;P62195;Q8WUM0;Q96HA1;P49792;P46060;O43264;O14980;Q86Y07-2;O75694;P30153;P36873;Q14739;Q99460;P50402;Q8WUX9;Q96FJ2;Q9UQE7;Q86XL3;Q92621;Q8WYP5;Q5SRE5;Q15691;Q16401
Mitotic Metaphase and Anaphase	35	238	0.038834501	Q9Y2U8;Q9Y266;Q14008;P42167-1;Q9BTX1;Q9H444;Q9UQN3;O43237;P51665;P35998;P52948-5;Q9Y6G9;P62195;Q8WUM0;Q96HA1;P49792;P46060;O43264;O14980;Q86Y07-2;O75694;P30153;P36873;Q14739;Q99460;P50402;Q8WUX9;Q96FJ2;Q9UQE7;Q86XL3;Q92621;Q8WYP5;Q5SRE5;Q15691;Q16401
RHOA GTPase cycle	33	150	1.16E-04	Q9H2G2;Q2M1Z3;Q15836;Q8WVM8;Q8N5G2;Q12802;Q03135;O95210;Q8NBN3;P42167;Q9UDY2;O15498;Q8IWB9;Q14254;P27105;O15173;Q07021;Q96HY6;P49257;Q16512;Q16513;Q5T5U3;Q14739;Q12979;Q9NSV4;Q07960;O60610;Q96RT1;O00161;Q9Y4D1;Q13190;Q13393;P43121
Rab regulation of trafficking	31	123	1.62E-05	Q5VZ89;Q9BYX2;Q9H2M9;P48553;Q7Z401;Q8TC07;Q96CV9;Q14C86;P61020;Q7Z392;P20339;Q8IWF6;Q8TEH3;Q9UJY5;Q15042;Q9UJY4;Q92574;Q8IUR0;Q8TBN0;P51151;P51153;P31749;Q96NW4;Q15907;P51159;Q9Y3P9;Q9Y2L5;P86790;Q8WVT3;Q4ADV7;Q15276
Golgi-to-ER retrograde transport	30	137	2.50E-04	Q14203;Q9UBF2;Q92538;Q9H2M9;O43731;Q9NP61;P33176;Q9P299;O43237;Q96L93;P84085;Q9Y6G9;Q9NZ43;Q9UJW0;Q9H0B6;P48444;Q15042;Q6NUQ1;Q15102;A2RRP1;O43264;Q9NRZ7;Q9P2W9;O75396;Q9NQT8;Q96G01;Q96FJ2;O14579;Q8N6T3;P46459
Mitotic Prophase	29	119	5.38E-05	P52948-3;P52948-4;Q9Y2U8;Q92539;O95476;P42167-1;Q9BTX1;Q9H8Y8;P35658;P78406;P52948-5;Q99567;O60763;Q8WUM0;Q96HA1;Q14693;P49792;O95347;P49790;Q66LE6;Q86Y07-2;O75694;P30153;Q8TD19;P50402;Q92621;Q5SRE5;Q9H2G9;O15504
Disorders of transmembrane transporters	29	181	0.022491213	P52948-3;P52948-4;Q9BTX1;P35658;P11166;P51665;O00400;P35613;P35998;P78406;P52948-5;Q99567;P62195;Q9GZP9;Q9NRA2;Q8WUM0;Q9UHW9;Q96HA1;P49792;P49790;O75477;O75694;Q08357;Q99460;Q92621;Q5SRE5;Q16401;O15504;Q6P1M0
RAC2 GTPase cycle	28	88	5.98E-07	Q9Y2U8;Q14126;Q15836;Q9Y6W5;Q03135;O95210;Q9Y2A7;P42167;Q86Y07;Q9UH65;O15173;P05556;Q96BY6;P49257;Q6IAA8;Q8IZP0;Q5T5U3;Q14739;Q12979;Q9NSV4;Q07960;P50402;Q96RT1;Q86XL3;P43121;Q13177;P29317;P42336
Host Interactions of HIV factors	28	144	0.002230135	P52948-3;P52948-4;Q10567;Q9BTX1;P35658;P51665;P35998;P78406;P52948-5;P12235;Q99567;P62195;Q8WUM0;Q96HA1;P49792;O94973;P46060;P49790;O14980;O75694;Q99460;O43747;Q92621;Q5SRE5;Q16401;Q13177;O15504;P43487
COPI-mediated anterograde transport	26	102	6.31E-05	Q14203;Q9UBF2;Q92538;O43731;Q9NP61;Q14746;Q14789;Q9P299;O43237;P84085;Q9Y6G9;O60763;Q9UJW0;Q8WTW3;P48444;O15498;P83436;Q9NRC6;Q12893;O15155;Q96FJ2;Q13190;Q96JB2;O14579;Q8N6T3;P46459
Cargo recognition for clathrin-mediated endocytosis	26	105	9.95E-05	P25098;P52594;Q96D71;Q15836;O75886;Q2M2I8;Q8WXE9;Q9Y6I3;Q5SW96;P51809;Q99961;Q0JRZ9;Q9NZM3;P98082;P61201;O14964;P42566;P11717;O94973;Q9BT88;Q92905;Q9UBC2;Q9NVZ3;Q13492;P00533;Q92783
CDC42 GTPase cycle	26	155	0.018122082	A4D1P6;Q2M1Z3;Q9NP61;Q15836;Q03135;Q5VT25;Q96HP0;O00401;P42167;Q96N67;O15498;P27105;Q96BY6;Q6IAA8;Q5T5U3;Q14739;Q12979;Q9NSV4;Q07960;O00161;Q9Y4D1;Q13393;Q13177;Q14344;Q15311;Q16584
trans-Golgi Network Vesicle Budding	25	74	8.26E-07	Q92538;Q10567;Q14789;O75146;O95295;Q12846;O00443;O60749;P55327;P51809;Q92572;Q0IIM8;Q9Y5X1;Q9Y5X3;O14964;Q01968;P11717;Q14677;O75976;Q9H3P7;Q9UPM8;O43747;O00161;Q13492;O14976
Late Phase of HIV Life Cycle	24	149	0.033502895	P49792;P52948-3;P09958;P46060;P52948-4;P49790;Q8NEZ2;O14980;Q9BTX1;Q9H444;Q9UQN3;O75694;P35658;O75351;P78406;P52948-5;Q8WUX9;Q99567;Q92621;Q5SRE5;O15504;P43487;Q8WUM0;Q96HA1
Nuclear Envelope Breakdown	23	58	1.53E-07	P49792;P52948-3;Q9Y2U8;P52948-4;Q92539;O95476;P49790;P42167-1;Q9BTX1;Q86Y07-2;O75694;Q8TD19;P35658;P78406;P52948-5;P50402;Q99567;Q92621;Q5SRE5;O15504;Q8WUM0;Q14693;Q96HA1
RHOC GTPase cycle	23	75	1.04E-05	Q9H2G2;Q07021;P49257;Q16512;Q16513;Q15836;Q8N5G2;Q5T5U3;Q12802;Q14739;Q03135;Q12979;Q9NSV4;Q07960;O60610;P42167;Q9UDY2;Q96RT1;Q9Y4D1;Q13190;P43121;Q14254;P27105
Antiviral mechanism by IFN-stimulated genes	23	89	1.34E-04	P49792;P52948-3;O95786;P52948-4;O00505;Q14258;P49790;Q9BTX1;Q9Y6K5;O75694;P35658;P78406;P52948-5;Q99567;P61221;Q92621;P29728;Q5SRE5;O15504;Q8WUM0;P06730;Q96HA1;P21333
Glucose metabolism	23	98	5.05E-04	P49792;P52948-3;P52948-4;O75746;P49790;Q16822;Q9BRR6;Q9BTX1;O75694;P30153;P35658;P78406;P52948-5;P53007;P18669;Q99567;Q92621;Q9UJS0;Q5SRE5;Q01813;O15504;Q8WUM0;Q96HA1
Retrograde transport at the Trans-Golgi-Network	22	49	3.49E-08	Q92805;P11717;Q9UIL1;P53365;P51151;Q15836;O43752;Q14746;Q9UID3;Q9P1Q0;Q8IWJ2;Q5VIR6;P82094;Q4ADV7;O60664;Q96CN9;O60499;Q8WTW3;P40616;Q96JB2;P83436;P46459
RAB GEFs exchange GTP for GDP on RABs	22	90	3.89E-04	Q8IUR0;Q5VZ89;Q9H2M9;Q8TBN0;P51151;P48553;P51153;P31749;Q7Z401;Q96NW4;P51159;Q14C86;Q9Y2L5;P61020;P20339;Q7Z392;P86790;Q8WVT3;Q4ADV7;Q8IWF6;Q8TEH3;Q15042
SLC transporter disorders	22	103	0.002115185	P49792;P52948-3;P52948-4;P49790;Q9BTX1;O75694;Q08357;P35658;P11166;O00400;P35613;P78406;P52948-5;Q99567;Q92621;Q5SRE5;O15504;Q9NRA2;Q9UHW9;Q8WUM0;Q96HA1;Q6P1M0
Golgi Associated Vesicle Biogenesis	21	56	1.24E-06	P11717;Q14677;O75976;Q14789;Q9H3P7;Q10567;O75146;O95295;O60749;O00443;Q9UPM8;P55327;O43747;P51809;Q0IIM8;Q92572;Q9Y5X1;Q13492;Q9Y5X3;Q01968;O14976
COPII-mediated vesicle transport	21	68	2.24E-05	Q8IUR0;Q9Y6Y8;O95487;O15084;O94855;O15155;P49257;P48553;Q8WVM8;Q12907;O95486;O75396;Q9UPN7;O60763;Q13190;O15027;O15498;Q9Y6B6;Q92734;Q9HCU5;P46459
Cellular response to heat stress	20	95	0.0038319	P49792;P52948-3;P52948-4;P49790;P54652;Q13315;Q13557;Q9BTX1;O75694;P35658;P78406;P52948-5;Q99567;Q92621;O95817;Q5SRE5;O15504;P25685;Q8WUM0;Q96HA1
COPI-dependent Golgi-to-ER retrograde traffic	20	103	0.008884447	A2RRP1;O43264;Q9UBF2;Q92538;O43731;Q9NP61;P33176;Q9P299;Q9P2W9;Q96L93;O75396;Q9NQT8;P84085;Q9NZ43;Q9H0B6;P48444;Q6NUQ1;O14579;Q8N6T3;P46459
tRNA processing	20	113	0.021592871	P49792;P52948-3;P52948-4;P49790;Q08J23;Q9Y224;Q9BTX1;Q5VV42;O75694;P35658;P78406;P52948-5;Q99567;Q96Q11;Q92621;Q5SRE5;Q92499;O15504;Q8WUM0;Q96HA1
Regulation of HSF1-mediated heat shock response	19	75	6.20E-04	P49792;P52948-3;P52948-4;P49790;P54652;Q13315;Q9BTX1;O75694;P35658;P78406;P52948-5;Q99567;Q92621;O95817;Q5SRE5;O15504;P25685;Q8WUM0;Q96HA1
Nuclear Envelope (NE) Reassembly	19	78	9.75E-04	P46060;Q9Y2U8;P42167-1;Q9BTX1;Q86Y07-2;Q9H444;Q9UQN3;O75694;P30153;Q14739;P52948-5;P50402;Q8WUX9;Q86XL3;Q92621;Q8WYP5;Q5SRE5;Q8WUM0;Q96HA1
Glycolysis	19	78	9.75E-04	P49792;P52948-3;P52948-4;P49790;Q9BRR6;Q9BTX1;O75694;P30153;P35658;P78406;P52948-5;P18669;Q99567;Q92621;Q5SRE5;Q01813;O15504;Q8WUM0;Q96HA1
ISG15 antiviral mechanism	19	80	0.001298417	P49792;P52948-3;O95786;P52948-4;O00505;Q14258;P49790;Q9BTX1;O75694;P35658;P78406;P52948-5;Q99567;Q92621;Q5SRE5;O15504;Q8WUM0;P06730;Q96HA1
PI Metabolism	19	84	0.002223121	Q9BTU6;Q8TBX8;Q9Y217;O15056;Q13614;Q96QG7;Q08AM6;O43426;Q99570;Q9Y2I7;O00443;Q86T03;Q9Y2H2;P20339;Q96T51;Q01968;Q9HB20;P42336;O15327
Interactions of Rev with host cellular proteins	18	42	1.14E-06	P49792;P52948-3;P46060;P52948-4;P49790;O14980;Q9BTX1;O75694;P35658;P78406;P52948-5;Q99567;Q92621;Q5SRE5;O15504;P43487;Q8WUM0;Q96HA1
SUMOylation of DNA replication proteins	18	50	1.19E-05	P49792;P52948-3;P46060;P52948-4;P49790;Q9BTX1;O75694;P35658;P78406;P52948-5;P12004;P11387;Q99567;Q92621;Q5SRE5;O15504;Q8WUM0;Q96HA1
tRNA processing in the nucleus	18	62	1.77E-04	P49792;P52948-3;P52948-4;P49790;Q9Y224;Q9BTX1;O75694;P35658;P78406;P52948-5;Q99567;Q96Q11;Q92621;Q5SRE5;Q92499;O15504;Q8WUM0;Q96HA1
RHOB GTPase cycle	18	71	8.43E-04	Q9H2G2;Q16512;Q16513;Q15836;Q5T5U3;Q12802;Q9NSV4;Q03135;Q12979;Q07960;O60610;Q9UDY2;Q96RT1;O00161;Q9Y4D1;P43121;Q14254;P27105
Transport of Mature Transcript to Cytoplasm	18	87	0.00694245	P49792;P52948-3;P52948-4;P49790;Q9BTX1;Q13838;O75694;P35658;P78406;P52948-5;O00148;Q99567;Q92621;Q5SRE5;O15504;Q8WUM0;P06730;Q96HA1
Transport of Mature mRNA derived from an Intron-Containing Transcript	17	78	0.005255151	P49792;P52948-3;P52948-4;P49790;Q9BTX1;Q13838;O75694;P35658;P78406;P52948-5;O00148;Q99567;Q92621;Q5SRE5;O15504;Q8WUM0;Q96HA1
Transcriptional regulation by small RNAs	17	80	0.006666952	P49792;P52948-3;P52948-4;P49790;Q9UKV8;Q9UL18;Q9BTX1;O75694;P35658;P78406;P52948-5;Q99567;Q92621;Q5SRE5;O15504;Q8WUM0;Q96HA1
Transport of the SLBP independent Mature mRNA	16	39	7.49E-06	P49792;P52948-3;P52948-4;P49790;Q9BTX1;O75694;P35658;P78406;P52948-5;Q99567;Q92621;Q5SRE5;O15504;Q8WUM0;P06730;Q96HA1
Transport of the SLBP Dependant Mature mRNA	16	40	1.02E-05	P49792;P52948-3;P52948-4;P49790;Q9BTX1;O75694;P35658;P78406;P52948-5;Q99567;Q92621;Q5SRE5;O15504;Q8WUM0;P06730;Q96HA1
Nuclear Pore Complex (NPC) Disassembly	16	40	1.02E-05	P49792;P52948-3;P52948-4;P49790;Q9BTX1;O75694;Q8TD19;P35658;P78406;P52948-5;Q99567;Q92621;Q5SRE5;O15504;Q8WUM0;Q96HA1
NEP/NS2 Interacts with the Cellular Export Machinery	16	42	1.83E-05	P49792;P52948-3;P52948-4;P49790;O14980;Q9BTX1;O75694;P35658;P78406;P52948-5;Q99567;Q92621;Q5SRE5;O15504;Q8WUM0;Q96HA1
Export of Viral Ribonucleoproteins from Nucleus	16	43	2.42E-05	P49792;P52948-3;P52948-4;P49790;O14980;Q9BTX1;O75694;P35658;P78406;P52948-5;Q99567;Q92621;Q5SRE5;O15504;Q8WUM0;Q96HA1
Transport of Mature mRNA Derived from an Intronless Transcript	16	46	5.31E-05	P49792;P52948-3;P52948-4;P49790;Q9BTX1;O75694;P35658;P78406;P52948-5;Q99567;Q92621;Q5SRE5;O15504;Q8WUM0;P06730;Q96HA1
NS1 Mediated Effects on Host Pathways	16	46	5.31E-05	P49792;P52948-3;P52948-4;O00505;P49790;Q9BTX1;O75694;P35658;P78406;P52948-5;Q99567;Q92621;Q5SRE5;O15504;Q8WUM0;Q96HA1
Transport of Mature mRNAs Derived from Intronless Transcripts	16	47	6.79E-05	P49792;P52948-3;P52948-4;P49790;Q9BTX1;O75694;P35658;P78406;P52948-5;Q99567;Q92621;Q5SRE5;O15504;Q8WUM0;P06730;Q96HA1
Interactions of Vpr with host cellular proteins	16	47	6.79E-05	P49792;P52948-3;P52948-4;P49790;Q9BTX1;O75694;P35658;P78406;P12235;P52948-5;Q99567;Q92621;Q5SRE5;O15504;Q8WUM0;Q96HA1
snRNP Assembly	16	58	6.75E-04	P49792;P52948-3;P52948-4;P49790;Q9BTX1;P14678;O75694;P35658;P78406;P52948-5;Q99567;Q92621;Q5SRE5;O15504;Q8WUM0;Q96HA1
Metabolism of non-coding RNA	16	58	6.75E-04	P49792;P52948-3;P52948-4;P49790;Q9BTX1;P14678;O75694;P35658;P78406;P52948-5;Q99567;Q92621;Q5SRE5;O15504;Q8WUM0;Q96HA1
Signaling by MET	16	80	0.013982878	P05556;Q68CZ2;P40818;P18031;O75886;P12931;P17301;P46109;Q96N67;Q99961;Q92783;O14964;P40763;P42566;P26006;P42336
SUMOylation of DNA damage response and repair proteins	16	81	0.01550672	P49792;P52948-3;P52948-4;P49790;Q9BTX1;O75694;P35658;P78406;P52948-5;Q99567;Q9UQE7;Q92621;Q5SRE5;O15504;Q8WUM0;Q96HA1
Defective TPR may confer susceptibility towards thyroid papillary carcinoma (TPC)	15	36	1.19E-05	P49792;P52948-3;P52948-4;P49790;Q9BTX1;O75694;P35658;P78406;P52948-5;Q99567;Q92621;Q5SRE5;O15504;Q8WUM0;Q96HA1
Regulation of Glucokinase by Glucokinase Regulatory Protein	15	36	1.19E-05	P49792;P52948-3;P52948-4;P49790;Q9BTX1;O75694;P35658;P78406;P52948-5;Q99567;Q92621;Q5SRE5;O15504;Q8WUM0;Q96HA1
SUMOylation of SUMOylation proteins	15	39	2.96E-05	P49792;P52948-3;P52948-4;P49790;Q9BTX1;O75694;P35658;P78406;P52948-5;Q99567;Q92621;Q5SRE5;O15504;Q8WUM0;Q96HA1
Nuclear import of Rev protein	15	39	2.96E-05	P49792;P52948-3;P52948-4;P49790;Q9BTX1;O75694;P35658;P78406;P52948-5;Q99567;Q92621;Q5SRE5;O15504;Q8WUM0;Q96HA1
Transport of Ribonucleoproteins into the Host Nucleus	15	40	3.93E-05	P49792;P52948-3;P52948-4;P49790;Q9BTX1;O75694;P35658;P78406;P52948-5;Q99567;Q92621;Q5SRE5;O15504;Q8WUM0;Q96HA1
SUMOylation of ubiquitinylation proteins	15	43	8.69E-05	P49792;P52948-3;P52948-4;P49790;Q9BTX1;O75694;P35658;P78406;P52948-5;Q99567;Q92621;Q5SRE5;O15504;Q8WUM0;Q96HA1
Vpr-mediated nuclear import of PICs	15	44	1.11E-04	P49792;P52948-3;P52948-4;P49790;Q9BTX1;O75694;P35658;P78406;P52948-5;Q99567;Q92621;Q5SRE5;O15504;Q8WUM0;Q96HA1
SUMOylation of RNA binding proteins	15	51	5.18E-04	P49792;P52948-3;P52948-4;P49790;Q9BTX1;O75694;P35658;P78406;P52948-5;Q99567;Q92621;Q5SRE5;O15504;Q8WUM0;Q96HA1
Viral Messenger RNA Synthesis	15	52	6.29E-04	P49792;P52948-3;P52948-4;P49790;Q9BTX1;O75694;P35658;P78406;P52948-5;Q99567;Q92621;Q5SRE5;O15504;Q8WUM0;Q96HA1
SUMOylation of chromatin organization proteins	15	62	0.003364925	P49792;P52948-3;P52948-4;P49790;Q9BTX1;O75694;P35658;P78406;P52948-5;Q99567;Q92621;Q5SRE5;O15504;Q8WUM0;Q96HA1
Signaling by EGFR	14	52	0.001769601	P78536;Q9UBC2;O75886;Q9Y6I3;P12931;Q05209;Q99961;P12931-1;P41240;Q92783;P00533;O14964;P42566;P42336
Synthesis of PIPs at the plasma membrane	14	53	0.002099549	Q9BTU6;Q8TBX8;Q9Y217;O15056;Q13614;Q96QG7;O43426;O00443;P20339;Q96T51;Q01968;Q9HB20;P42336;O15327
RHOQ GTPase cycle	14	59	0.005314875	Q9NXC2;Q15836;Q6IAA8;Q5T5U3;Q9NSV4;Q03135;Q07960;Q5VT25;O00401;O00161;Q9HD26;Q13177;Q15642;P27105
RHOF GTPase cycle	13	42	7.53E-04	Q15836;P12814;Q5T5U3;Q9NSV4;Q03135;Q07960;O60610;P42167;O00161;Q5JTV8;Q02952;P43121;P80723
RHOJ GTPase cycle	13	55	0.007280609	Q15836;Q6IAA8;Q5T5U3;Q9NSV4;Q03135;Q07960;Q5VT25;O00401;P42167;O00161;Q13177;Q01968;P27105
Signaling by ALK fusions and activated point mutants	13	57	0.009588898	P49792;Q14203;Q86W92;P33176;O43815;Q8IWJ2;O00291;P17275;Q9NR09;Q92734;P40763;Q9NZJ5;P42336
Signaling by ALK in cancer	13	57	0.009588898	P49792;Q14203;Q86W92;P33176;O43815;Q8IWJ2;O00291;P17275;Q9NR09;Q92734;P40763;Q9NZJ5;P42336
RHOH GTPase cycle	12	37	7.98E-04	Q9BXS4;Q15836;Q9BZH6;P41240;Q6IAA8;Q9BZF9;Q658Y4;Q99707;Q03135;Q9BXB4;Q13177;P27105
Intra-Golgi traffic	12	44	0.003285666	O95721;Q15643;Q4ADV7;O43752;Q14746;Q8WTW3;Q13190;Q96JB2;O15498;P83436;Q8TBA6;P46459
TBC/RABGAPs	11	45	0.010268926	P61020;P20339;Q9BYX2;Q15907;Q8TC07;Q9UJY5;Q9UJY4;Q92574;Q96CV9;Q9Y3P9;Q15276
TGF-beta receptor signaling activates SMADs	11	46	0.011914504	P09958;P05556;Q9Y4E8;Q9Y3F4;P62140;P18084;O95405-1;O14980;P36873;Q15796;P62136
Cargo concentration in the ER	10	33	0.003395736	O95487;O94855;P49257;Q5JRA6;Q13190;Q9Y6B6;Q12907;O95486;O75396;Q9HCU5
RHOU GTPase cycle	10	40	0.012143358	Q2M1Z3;O75886;Q9NZM3;P12931;Q92783;Q13177;O14964;P29317;Q9H792;Q93008
Postmitotic nuclear pore complex (NPC) reformation	9	28	0.003648461	P46060;Q8WYP5;Q92621;Q9BTX1;Q5SRE5;O75694;Q8WUM0;P52948-5;Q96HA1
EGFR downregulation	9	31	0.006916869	Q05209;Q99961;Q9UBC2;O75886;Q9Y6I3;P00533;Q92783;O14964;P42566
MTOR signalling	9	41	0.034406824	Q9Y2Q5;Q9HB90;Q5VZM2;Q6IAA8;P31749;Q92574;Q9UHA4;Q13131;P06730
RND1 GTPase cycle	9	42	0.039047682	Q9Y2U8;Q9ULH0;Q9BXS4;O75976;Q9UJF2;Q03135;P51648;P29317;Q14254
RND2 GTPase cycle	9	43	0.044096151	Q9Y2U8;A0JNW5;Q9ULH0;Q9Y266;Q03135;P51648;P29317;Q08378;Q07960
Synthesis of PIPs at the early endosome membrane	8	16	3.92E-04	Q9BTU6;Q13614;Q08AM6;Q99570;Q9Y2I7;O00443;O15327;Q9Y2H2
Downregulation of TGF-beta receptor signaling	8	26	0.007631422	Q9Y4E8;Q9Y3F4;P62140;O95405-1;O14980;P36873;Q15796;P62136
Endosomal Sorting Complex Required For Transport (ESCRT)	8	31	0.019831095	Q8WUX9;Q8NEZ2;O75886;Q9H444;Q9UQN3;Q92783;O75351;O14964
Initiation of Nuclear Envelope (NE) Reformation	7	20	0.006312074	Q9Y2U8;Q86XL3;Q86Y07-2;P42167-1;P30153;Q14739;P50402
Negative regulation of MET activity	7	21	0.008120348	P40818;Q99961;P18031;O75886;Q92783;O14964;P42566
Insulin processing	7	27	0.027656043	O60645;Q8TAD4;Q8NEW0;Q96A65;Q96HE7;P33176;P51159
Energy dependent regulation of mTOR by LKB1-AMPK	7	29	0.038234964	Q9Y2Q5;Q9HB90;Q5VZM2;Q6IAA8;Q92574;Q9UHA4;Q13131
Synthesis of PIPs at the late endosome membrane	6	11	0.00133022	Q13614;Q96QG7;Q08AM6;Q99570;Q9Y2I7;O00443
SARS-CoV-2 modulates autophagy	6	14	0.004333258	Q9H270;Q9H269;Q9P253;Q96AX1;Q9H267;Q96JC1
Depolymerisation of the Nuclear Lamina	6	16	0.008094881	Q9Y2U8;Q92539;O95476;P42167-1;P50402;Q14693
Synthesis of PIPs at the Golgi membrane	6	18	0.013770559	Q9BTU6;Q08AM6;Q99570;Q9Y2I7;O00443;Q01968
RHO GTPases activate PAKs	6	21	0.026737204	Q14247;P62140;P35240;Q13177;O14974;P21333
Listeria monocytogenes entry into host cells	6	23	0.03881559	Q99961;O75886;P12931;Q92783;O14964;P42566
mTORC1-mediated signalling	6	24	0.045971246	Q9Y2Q5;Q9HB90;Q5VZM2;Q6IAA8;Q9UHA4;P06730
OAS antiviral response	5	10	0.004764289	O95786;P61221;P29728;Q9Y6K5;P21333
Synthesis of IP2, IP, and Ins in the cytosol	5	14	0.018193645	P32019;Q96QG7;O43426;Q01968;O15327
InlB-mediated entry of Listeria monocytogenes into host cell	5	17	0.037260998	Q99961;O75886;Q92783;O14964;P42566
Cation-coupled Chloride cotransporters	4	7	0.007159706	Q9Y666;P55011;Q9UP95;Q9UHW9
ALK mutants bind TKIs	4	12	0.041039401	O00291;Q9NR09;O43815;Q9NZJ5
Post-transcriptional silencing by small RNAs	3	7	0.040013952	Q9UKV8;Q9H9G7;Q9UL18
Defective MTR causes HMAG	2	2	0.019752354	Q9UBK8;Q99707
Defective MTRR causes HMAE	2	2	0.019752354	Q9UBK8;Q99707
