# Supplementary material for: Proximity-dependent mapping of the HCMV US28 interactome identifies RhoGEF signaling as a requirement for efficient viral reactivation
Source: PLoS Pathog. 2023 Oct 2;19(10):e1011682. doi: 10.1371/journal.ppat.1011682 (PMC10569644; doi:10.1371/journal.ppat.1011682)
Supplement: S3 Table — (PDF) [file ppat.1011682.s006.pdf]

Supplemental Information Table S3. Pathway Analysis of HCMV-US28 BirA Interactome in CD34+ HPCs

Pathway Name	#Entities found	#Entities total	Entities pValue	Mapped Entities
Neutrophil degranulation	85	480	2.91E-11	Q13835;P28482;Q92619;P04839;P39656;P51665;O95352;P51149;P61020;Q70J99;Q02413;P63000;P01833;O15511;P04040;P61225;P09972;P61224;P46940;O95716;P41218;P61586;P09211;P00387;P27105;Q96CS3;Q07065;P06702;Q9NP72;Q9BS26;Q14019;Q92608;O00231;P11216;O75131;Q08554;P18206;Q9NZT1;P84095;P35573;P14174;Q92882;P01024;Q01518;P50395;P02792;Q13576;P13727;P05109;Q9Y2Y8;Q13217;P29350;Q9P0L0;O75165;O00487;O00764;P30740;Q01469;P61160;Q7Z6Z7;P29508;O94804;Q9UJU6;P11717;Q15008;Q5T4S7;Q8TD55;O75874;P11678;Q96P63;P17931;Q99536;P68402;O60234;P17858;Q05655;Q5D862;P09960;P31949;P07384;P20340;P05089;P07741;Q92820;Q14165
Signaling by Rho GTPases	68	678	0.041161295	P28482;Q14008;Q92619;P04839;P12931;Q9P2R3;P52948-5;P51149;Q06187;P62993-1;P12931-1;Q02413;P63000;O15511;P62714;P43034;P46940;P61586;P05771;P62995;Q14254;Q8WUM0;P27105;Q96CS3;Q9Y512;P52565;Q07065;P06702;O95202;Q16512;P49257;P27361;Q15746;Q92608;Q16718;P59998;P52566;P84095;P42331;P41240;P11182;Q8WYP5;P61158;Q9BSJ8;Q92888;Q13576;O15144;O15145;P05109;O75489;O15143;O75323;P57740;O95292;Q15080;Q9Y2A7;P61160;P15153;O94804;P61201;Q9BX68;Q93008;O43264;Q07960;P19878;Q8N392;Q05655;Q15691
Platelet activation, signaling and aggregation	42	265	4.21E-05	P08758;Q15833;P08514;P05106;P04899;O43852;P28482;P12259;P29350;P12931;P13224;O00194;P15153;P62993-1;P12931-1;P63000;Q9NTK5;P61224;P00488;P61586;P08567;O15439;P05771;P07996;Q9UHQ9;Q8NBX0;Q86UX7;P27361;Q7Z5R6;P16885;O75083;P18206;P84095;P43405;Q05655;P00734;P41240;P08754;P49407;Q8NCG7;P02751;Q01518
Metabolism of carbohydrates	37	300	0.008551268	P52948-3;P52948-4;O43252;P13807;P37837;P40926;Q16822;Q9BRR6;Q8TEM1;P57740;Q3LXA3;P35658;O60825;P52948-5;Q00796;P60174;P09972;P62714;Q14376;Q8WUM0;P29401;P51570;P52209;O75694;P11216;P11413;P19367;P14550;P15121;P35573;Q04446;P17858;Q99653;P00558;Q92621;P08237;Q01813
Cellular response to chemical stress	33	201	1.45E-04	P37837;P30043;P04839;Q06323;P51665;O00487;Q15080;P48507;P13073;P04040;Q96HE7;P09211;P69905;P40763;P25786;Q15008;P29401;Q8TAT6;P30044-1;O75874;P52209;O00231;P30044-2;P62191;P11413;Q86X55;P19878;O14818;Q05655;Q16401;P43686;P07203;P04179
The citric acid (TCA) cycle and respiratory electron transport	28	178	8.45E-04	P31930;O75947;O75306;P08559;P13804;P40926;O75489;Q04760;P50213;O43837;Q9UDW1;P13073;P24539;P31040;P23368;Q16795;P07954;Q13423;P53597;P36957;P48047;P28331;P11177;Q16718;P38117;P47985;P21796;P48735
Fatty acid metabolism	25	179	0.006885541	P22307-1;Q9BV79;Q9P035;P50416;P33897;Q53GQ0;P42765;O60760;P23786;Q86TX2;Q4G176;Q16698;Q16836;P51659;P22307;Q9NPJ3;P23219;P16152;P30084;P09960;P16050;P55084;P49748;Q15067-2;P07203
Disorders of transmembrane transporters	23	181	0.02460382	Q15008;P52948-3;P52948-4;Q8TEM1;O00231;P57740;O75477;P62191;O75694;Q06323;P35658;P33897;P51665;P19367;O00487;Q9NP58;P52948-5;O14818;Q92621;Q16401;P43686;Q8WUM0;P25786
Separation of Sister Chromatids	23	191	0.040873509	Q15008;O43264;Q14008;O00231;P57740;P62191;Q06323;P51665;O00487;P52948-5;O14818;Q9UQE7;Q8WYP5;P62714;Q9NTI5;P43034;Q8N3U4;Q15691;Q16401;P43686;Q8WUM0;Q29RF7;P25786
Fcgamma receptor (FCGR) dependent phagocytosis	22	175	0.030348834	O15144;O15145;P28482;P27361;O15143;Q8IV08;P0DOY2;P59998;Q06187;P16885;O00159;P43405;Q9Y2A7;P61160;P62993-1;Q05655;P12931-1;P63000;P01834;O15511;P61158;Q92556
Glucose metabolism	21	98	7.41E-05	P52948-3;P52948-4;P40926;Q16822;Q9BRR6;Q8TEM1;P57740;O75694;P35658;O60825;P19367;P52948-5;P17858;P00558;P60174;Q92621;P09972;P62714;P08237;Q01813;Q8WUM0
Antiviral mechanism by IFN-stimulated genes	20	89	5.80E-05	P52948-3;P09914;P52948-4;Q14258;P20591;P27361;O00629;Q8TEM1;P57740;O75694;P35658;P52948-5;P42224;P61221;P05161;Q92621;P29728;Q8WUM0;P41226;O14933
Response to elevated platelet cytosolic Ca2+	20	133	0.006970817	Q9UHQ9;P08758;Q15833;Q8NBX0;P08514;Q86UX7;P05106;O43852;P12259;O75083;P18206;O00194;Q9NTK5;P00488;P08567;O15439;P05771;P07996;Q01518;P02751
Glycolysis	19	78	3.08E-05	P52948-3;P52948-4;Q9BRR6;Q8TEM1;P57740;O75694;P35658;O60825;P19367;P52948-5;P17858;P00558;P60174;Q92621;P09972;P62714;P08237;Q01813;Q8WUM0
Mitotic Prophase	19	119	0.004594893	P52948-3;O95347;P52948-4;P28482;P27361-3;Q8TEM1;P57740;Q9H8Y8;O75694;P35658;P52948-5;Q9H0U4;O60763;Q92621;P62714;P05771;P61019;P62820;Q8WUM0
Platelet degranulation	19	128	0.009515808	Q9UHQ9;P08758;Q15833;Q8NBX0;P08514;Q86UX7;P05106;O43852;P12259;O75083;P18206;O00194;Q9NTK5;P00488;P08567;O15439;P07996;Q01518;P02751
Leishmania phagocytosis	19	149	0.037207719	O15144;O15145;P28482;P27361;O15143;P0DOY2;P59998;Q06187;O00159;P43405;Q9Y2A7;P61160;P62993-1;P12931-1;P63000;P01834;O15511;P61158;Q92556
FCGR3A-mediated phagocytosis	19	149	0.037207719	O15144;O15145;P28482;P27361;O15143;P0DOY2;P59998;Q06187;O00159;P43405;Q9Y2A7;P61160;P62993-1;P12931-1;P63000;P01834;O15511;P61158;Q92556
Parasite infection	19	149	0.037207719	O15144;O15145;P28482;P27361;O15143;P0DOY2;P59998;Q06187;O00159;P43405;Q9Y2A7;P61160;P62993-1;P12931-1;P63000;P01834;O15511;P61158;Q92556
ISG15 antiviral mechanism	18	80	1.31E-04	P52948-3;P09914;P52948-4;Q14258;P20591;P27361;O00629;Q8TEM1;P57740;O75694;P35658;P52948-5;P42224;P05161;Q92621;Q8WUM0;P41226;O14933
KEAP1-NFE2L2 pathway	18	108	0.003719236	Q15008;P29401;Q8TAT6;P37837;O75874;P52209;O00231;P62191;Q06323;P11413;P51665;O00487;P48507;O14818;Q05655;Q16401;P43686;P25786
PTEN Regulation	18	139	0.036889945	Q15008;Q9HB90;Q9UL18;O94776;P28482;P27361;O00231;P62191;P29590;Q06323;P51665;O00487;P42345;O14818;Q16401;Q93009;P43686;P25786
Signaling by BRAF and RAF1 fusions	17	66	4.04E-05	O43252;P08514;P05106;P28482;P27361;P12931;Q02750;Q7Z5R6;O00203;O95352;P18206;Q7Z2W4;P41240;P46940;P61224;P49407;P02751
Oncogenic MAPK signaling	17	84	6.48E-04	O43252;P08514;P05106;P28482;P27361;P12931;Q02750;Q7Z5R6;O00203;O95352;P18206;Q7Z2W4;P41240;P46940;P61224;P49407;P02751
Rab regulation of trafficking	17	123	0.024911787	Q9NVG8;P50395;Q9UL25;Q9H2M9;Q13637;Q9NP72;P51153;P51149;P62330;P61020;Q9H0U4;P62491;O00194;P61006;Q9UJY4;P20340;P62820
Nuclear events mediated by NFE2L2	16	79	9.14E-04	Q15008;P29401;P37837;O75874;P52209;O00231;P62191;Q06323;P11413;P51665;O00487;P48507;O14818;Q16401;P43686;P25786
PCP/CE pathway	16	92	0.004060824	Q15008;O00231;P62191;Q06323;P51665;O00487;O95782;P15153;O14818;P63000;Q96CW1;Q16401;P61586;P05771;P43686;P25786
Regulation of mRNA stability by proteins that bind AU-rich ele	15	88	0.00630014	Q15008;Q9Y2L1;O00231;P62191;Q06323;P35658;P51665;O00487;O14818;Q05655;Q92973;Q16401;P04792;P43686;P25786
MAPK6/MAPK4 signaling	15	89	0.006944454	Q15008;Q9UL18;O00231;P62191;Q06323;P51665;O00487;Q16181;O14818;P63000;Q16401;P04792;P25685;P43686;P25786
Cellular response to heat stress	15	95	0.011997747	P52948-3;P52948-4;P28482;P27361;Q8TEM1;P57740;O75694;P35244;P35658;P52948-5;Q02790;P42345;Q92621;P25685;Q8WUM0
CLEC7A (Dectin-1) signaling	15	98	0.015421464	Q15008;O00231;P62191;Q06323;P51665;O00487;P61081;P16885;P43405;O14818;Q05655;P12931-1;Q16401;P43686;P25786
ABC-family proteins mediated transport	15	103	0.02274291	Q15008;O00231;O75477;P62191;Q06323;P33897;P51665;O00487;Q9NP58;O14818;P20042;Q16401;O15439;P43686;P25786
Pyruvate metabolism and Citric Acid (TCA) cycle	14	55	2.11E-04	P07954;Q13423;P08559;P36957;P53597;P40926;Q04760;P50213;P11177;O43837;P31040;P23368;P21796;P48735
RAB geranylgeranylation	14	65	0.001056728	Q9UL25;Q13637;Q9NP72;P51153;P51149;P61020;Q9H0U4;P62491;O00194;P61006;O95716;P20340;P61019;P62820
RAC2 GTPase cycle	14	88	0.014028358	Q9Y512;P52565;P49257;Q92608;O95292;P04839;Q07960;P51149;Q15080;P19878;Q9Y2A7;P15153;P46940;Q9BSJ8
RHO GTPases Activate WASPs and WAVEs	13	36	1.11E-05	O15144;O15145;P28482;P27361;O15143;P59998;Q06187;Q9Y2A7;P61160;P62993-1;P63000;O15511;P61158
Signaling by high-kinase activity BRAF mutants	13	37	1.47E-05	P08514;P05106;P28482;P27361;P12931;Q02750;Q7Z5R6;P18206;P41240;P46940;P61224;P49407;P02751
MAP2K and MAPK activation	13	41	4.18E-05	P08514;P05106;P28482;P27361;P12931;Q02750;Q7Z5R6;P18206;P41240;P46940;P61224;P49407;P02751
Signaling by RAF1 mutants	13	42	5.31E-05	P08514;P05106;P28482;P27361;P12931;Q02750;Q7Z5R6;P18206;P41240;P46940;P61224;P49407;P02751
Signaling by moderate kinase activity BRAF mutants	13	47	1.59E-04	P08514;P05106;P28482;P27361;P12931;Q02750;Q7Z5R6;P18206;P41240;P46940;P61224;P49407;P02751
Paradoxical activation of RAF signaling by kinase inactive BRA	13	47	1.59E-04	P08514;P05106;P28482;P27361;P12931;Q02750;Q7Z5R6;P18206;P41240;P46940;P61224;P49407;P02751
Signaling downstream of RAS mutants	13	47	1.59E-04	P08514;P05106;P28482;P27361;P12931;Q02750;Q7Z5R6;P18206;P41240;P46940;P61224;P49407;P02751
Signaling by RAS mutants	13	47	1.59E-04	P08514;P05106;P28482;P27361;P12931;Q02750;Q7Z5R6;P18206;P41240;P46940;P61224;P49407;P02751
RHOG GTPase cycle	13	74	0.008338165	P52565;Q13576;O95202;P49257;O75489;Q92608;O95292;Q16718;Q07960;P52566;P51149;P84095;Q9BSJ8
Regulation of HSF1-mediated heat shock response	13	75	0.009244157	P52948-3;P52948-4;P28482;P27361;Q8TEM1;P57740;O75694;P35244;P35658;P52948-5;Q92621;P25685;Q8WUM0
ABC transporter disorders	13	78	0.012430098	Q15008;O00231;O75477;P62191;Q06323;P33897;P51665;O00487;Q9NP58;O14818;Q16401;P43686;P25786
RAB GEFs exchange GTP for GDP on RABs	13	90	0.034139144	P50395;Q9UL25;Q9H2M9;Q13637;Q9NP72;P51153;P51149;P61020;Q9H0U4;O00194;P61006;P20340;P62820
Platelet Aggregation (Plug Formation)	12	40	1.35E-04	P08514;P05106;P43405;P62993-1;P12931-1;P41240;P00734;P61224;P12931;P13224;Q7Z5R6;P02751
Vif-mediated degradation of APOBEC3G	12	56	0.002429348	Q15008;Q15369;Q15370;O14818;O00231;P62191;Q16401;Q06323;P51665;O00487;P43686;P25786
Oxygen-dependent proline hydroxylation of Hypoxia-inducible	12	66	0.008557573	Q15008;Q15369;Q15370;O14818;O00231;P62191;Q16401;Q06323;P51665;O00487;P43686;P25786
RHOC GTPase cycle	12	75	0.020997953	P52565;Q16512;P49257;Q8N392;O94804;O95292;P46940;P61586;Q07960;Q92888;Q14254;P27105
Cellular response to hypoxia	12	76	0.022941798	Q15008;Q15369;Q15370;O14818;O00231;P62191;Q16401;Q06323;P51665;O00487;P43686;P25786
SUMOylation of DNA damage response and repair proteins	12	81	0.034690852	P52948-3;P52948-4;Q9UQE7;Q92621;Q8TEM1;P57740;P29590;O75694;Q8N3U4;P35658;Q8WUM0;P52948-5
Degradation of beta-catenin by the destruction complex	12	83	0.04041521	Q15008;O14818;O00231;P62191;P62714;Q16401;Q06323;P51665;O00487;P43686;Q13363;P25786
RHO GTPases Activate NADPH Oxidases	11	24	5.97E-06	Q15080;P19878;P06702;P15153;Q05655;P05109;P28482;P63000;P27361;P04839;P05771
Transport of Mature mRNA Derived from an Intronless Transc	11	46	0.001577516	P52948-3;P52948-4;Q09161;Q92621;Q8TEM1;P57740;O75694;P35658;Q9UKF6;Q8WUM0;P52948-5
NS1 Mediated Effects on Host Pathways	11	46	0.001577516	P52948-3;P52948-4;O00629;P05161;Q92621;Q8TEM1;P57740;O75694;P35658;Q8WUM0;P52948-5
Transport of Mature mRNAs Derived from Intronless Transcri	11	47	0.001861216	P52948-3;P52948-4;Q09161;Q92621;Q8TEM1;P57740;O75694;P35658;Q9UKF6;Q8WUM0;P52948-5
Regulation of Apoptosis	11	53	0.004557874	Q15008;O60313;O14818;O00231;P62191;Q16401;Q06323;P51665;P43686;O00487;P25786
Regulation of RUNX3 expression and activity	11	55	0.005946414	Q15008;O14818;O00231;P62191;Q16401;P12931;Q06323;P51665;P43686;O00487;P25786
AUF1 (hnRNP D0) binds and destabilizes mRNA	11	56	0.006755257	Q15008;O14818;O00231;P62191;Q16401;Q06323;P04792;P51665;P43686;O00487;P25786
NIK-->noncanonical NF-kB signaling	11	59	0.009705782	Q15008;O14818;O00231;P62191;Q16401;Q06323;P51665;P43686;O00487;P61081;P25786
Metabolism of polyamines	11	59	0.009705782	Q15008;O14818;O00231;P62191;Q16401;Q06323;P51665;P43686;O00487;P19623;P25786
Dectin-1 mediated noncanonical NF-kB signaling	11	60	0.010882741	Q15008;O14818;O00231;P62191;Q16401;Q06323;P51665;P43686;O00487;P61081;P25786
Defective CFTR causes cystic fibrosis	11	61	0.012165969	Q15008;O14818;O00231;O75477;P62191;Q16401;Q06323;P51665;P43686;O00487;P25786
Activation of NF-kappaB in B cells	11	67	0.022418557	Q15008;O14818;O00231;P62191;Q16401;Q06323;P51665;P05771;P43686;O00487;P25786
Orc1 removal from chromatin	11	71	0.032092449	Q15008;O14818;O00231;P62191;Q16401;Q06323;P51665;P43686;O00487;P49736;P25786
Regulation of RUNX2 expression and activity	11	74	0.041069779	Q15008;P42224;O14818;O00231;P62191;Q16401;Q06323;P51665;P43686;O00487;P25786
Citric acid cycle (TCA cycle)	10	22	1.68E-05	P07954;Q13423;P36957;P53597;P40926;P31040;P23368;P50213;O43837;P48735
Integrin signaling	10	28	1.22E-04	P08514;P05106;P43405;P62993-1;P12931-1;P41240;P61224;P12931;Q7Z5R6;P02751
Transport of the SLBP independent Mature mRNA	10	39	0.001534018	P52948-3;P52948-4;Q09161;Q92621;Q8TEM1;P57740;O75694;P35658;Q8WUM0;P52948-5
Detoxification of Reactive Oxygen Species	10	39	0.001534018	Q15080;P19878;P30044-1;P04040;P30044-2;Q96HE7;P04839;P09211;P07203;P04179
Transport of the SLBP Dependant Mature mRNA	10	40	0.001841137	P52948-3;P52948-4;Q09161;Q92621;Q8TEM1;P57740;O75694;P35658;Q8WUM0;P52948-5
EPHB-mediated forward signaling	10	42	0.002604133	P61160;O15144;O15145;P12931-1;P63000;O15143;O15511;P61158;P61586;P59998
SUMOylation of ubiquitinylation proteins	10	43	0.003070706	P52948-3;P52948-4;Q92621;Q8TEM1;P57740;P29590;O75694;P35658;Q8WUM0;P52948-5
SUMOylation of DNA replication proteins	10	50	0.008486672	P52948-3;P52948-4;Q92621;Q8TEM1;P57740;O75694;P35658;P11388;Q8WUM0;P52948-5
Regulation of activated PAK-2p34 by proteasome mediated d	10	50	0.008486672	Q15008;O14818;O00231;P62191;Q16401;Q06323;P51665;P43686;O00487;P25786
Cross-presentation of soluble exogenous antigens (endosome	10	50	0.008486672	Q15008;O14818;O00231;P62191;Q16401;Q06323;P51665;P43686;O00487;P25786
Regulation of ornithine decarboxylase (ODC)	10	51	0.00964618	Q15008;O14818;O00231;P62191;Q16401;Q06323;P51665;P43686;O00487;P25786
Viral Messenger RNA Synthesis	10	52	0.0109228	P52948-3;P52948-4;Q92621;Q8TEM1;P57740;O75694;P19387;P35658;Q8WUM0;P52948-5
p53-Independent DNA Damage Response	10	52	0.0109228	Q15008;O14818;O00231;P62191;Q16401;Q06323;P51665;P43686;O00487;P25786
GSK3B and BTRC:CUL1-mediated-degradation of NFE2L2	10	52	0.0109228	Q15008;O14818;O00231;P62191;Q16401;Q06323;P51665;P43686;O00487;P25786
p53-Independent G1/S DNA damage checkpoint	10	52	0.0109228	Q15008;O14818;O00231;P62191;Q16401;Q06323;P51665;P43686;O00487;P25786
Ubiquitin Mediated Degradation of Phosphorylated Cdc25A	10	52	0.0109228	Q15008;O14818;O00231;P62191;Q16401;Q06323;P51665;P43686;O00487;P25786
Ubiquitin-dependent degradation of Cyclin D	10	52	0.0109228	Q15008;O14818;O00231;P62191;Q16401;Q06323;P51665;P43686;O00487;P25786
Autodegradation of the E3 ubiquitin ligase COP1	10	52	0.0109228	Q15008;O14818;O00231;P62191;Q16401;Q06323;P51665;P43686;O00487;P25786
Vpu mediated degradation of CD4	10	53	0.01232359	Q15008;O14818;O00231;P62191;Q16401;Q06323;P51665;P43686;O00487;P25786
FBXL7 down-regulates AURKA during mitotic entry and in earl	10	55	0.015525877	Q15008;O14818;O00231;P62191;Q16401;Q06323;P51665;P43686;O00487;P25786
SCF-beta-TrCP mediated degradation of Emi1	10	55	0.015525877	Q15008;O14818;O00231;P62191;Q16401;Q06323;P51665;P43686;O00487;P25786
Degradation of AXIN	10	55	0.015525877	Q15008;O14818;O00231;P62191;Q16401;Q06323;P51665;P43686;O00487;P25786
Negative regulation of NOTCH4 signaling	10	55	0.015525877	Q15008;O14818;O00231;P62191;Q16401;Q06323;P51665;P43686;O00487;P25786
Hh mutants are degraded by ERAD	10	56	0.017341352	Q15008;O14818;O00231;P62191;Q16401;Q06323;P51665;P43686;O00487;P25786
Signaling by ALK fusions and activated point mutants	10	57	0.019308889	P18206;P62993;P28482;P27361;Q9HC35;P31939;P10644;O43815;P02751;P40763
Signaling by ALK in cancer	10	57	0.019308889	P18206;P62993;P28482;P27361;Q9HC35;P31939;P10644;O43815;P02751;P40763
Degradation of DVL	10	57	0.019308889	Q15008;O14818;O00231;P62191;Q16401;Q06323;P51665;P43686;O00487;P25786
Stabilization of p53	10	57	0.019308889	Q15008;O14818;O00231;P62191;Q16401;Q06323;P51665;P43686;O00487;P25786
snRNP Assembly	10	58	0.021435207	P52948-3;P52948-4;Q09161;Q92621;Q8TEM1;P57740;O75694;P35658;Q8WUM0;P52948-5
Metabolism of non-coding RNA	10	58	0.021435207	P52948-3;P52948-4;Q09161;Q92621;Q8TEM1;P57740;O75694;P35658;Q8WUM0;P52948-5
Nuclear Envelope Breakdown	10	58	0.021435207	P52948-3;P52948-4;Q92621;Q8TEM1;P57740;O75694;P35658;P05771;Q8WUM0;P52948-5
Hh mutants abrogate ligand secretion	10	59	0.023726858	Q15008;O14818;O00231;P62191;Q16401;Q06323;P51665;P43686;O00487;P25786
Degradation of GLI1 by the proteasome	10	60	0.026190195	Q15008;O14818;O00231;P62191;Q16401;Q06323;P51665;P43686;O00487;P25786
GLI3 is processed to GLI3R by the proteasome	10	60	0.026190195	Q15008;O14818;O00231;P62191;Q16401;Q06323;P51665;P43686;O00487;P25786
Degradation of GLI2 by the proteasome	10	60	0.026190195	Q15008;O14818;O00231;P62191;Q16401;Q06323;P51665;P43686;O00487;P25786
SCF(Skp2)-mediated degradation of p27/p21	10	60	0.026190195	Q15008;O14818;O00231;P62191;Q16401;Q06323;P51665;P43686;O00487;P25786
Peroxisomal protein import	10	64	0.037878783	P35914;P22307;Q9Y2Q3;O75874;P04040;Q9BTZ2;P51659;O00116;Q93008;Q15067
Autodegradation of Cdh1 by Cdh1:APC/C	10	64	0.037878783	Q15008;O14818;O00231;P62191;Q16401;Q06323;P51665;P43686;O00487;P25786
Asymmetric localization of PCP proteins	10	64	0.037878783	Q15008;O14818;O00231;P62191;Q16401;Q06323;P51665;P43686;O00487;P25786
Hedgehog ligand biogenesis	10	65	0.041286758	Q15008;O14818;O00231;P62191;Q16401;Q06323;P51665;P43686;O00487;P25786
p53-Dependent G1 DNA Damage Response	10	66	0.044898694	Q15008;O14818;O00231;P62191;Q16401;Q06323;P51665;P43686;O00487;P25786
p53-Dependent G1/S DNA damage checkpoint	10	66	0.044898694	Q15008;O14818;O00231;P62191;Q16401;Q06323;P51665;P43686;O00487;P25786
Defective TPR may confer susceptibility towards thyroid papil	9	36	0.003049495	P52948-3;P52948-4;Q92621;Q8TEM1;P57740;O75694;P35658;Q8WUM0;P52948-5
Regulation of Glucokinase by Glucokinase Regulatory Protein	9	36	0.003049495	P52948-3;P52948-4;Q92621;Q8TEM1;P57740;O75694;P35658;Q8WUM0;P52948-5
Glutathione conjugation	9	37	0.003639157	P48507;P14550;Q96KP4;Q9Y2Q3;O60760;O14880;P21266;P09488;P09211
Mitochondrial Fatty Acid Beta-Oxidation	9	38	0.00431469	P42765;Q16836;Q9NPJ3;Q9BV79;P30084;Q86TX2;Q16698;P55084;P49748
SUMOylation of SUMOylation proteins	9	39	0.005084206	P52948-3;P52948-4;Q92621;Q8TEM1;P57740;O75694;P35658;Q8WUM0;P52948-5
Nuclear import of Rev protein	9	39	0.005084206	P52948-3;P52948-4;Q92621;Q8TEM1;P57740;O75694;P35658;Q8WUM0;P52948-5
Nuclear Pore Complex (NPC) Disassembly	9	40	0.005956059	P52948-3;P52948-4;Q92621;Q8TEM1;P57740;O75694;P35658;Q8WUM0;P52948-5
Transport of Ribonucleoproteins into the Host Nucleus	9	40	0.005956059	P52948-3;P52948-4;Q92621;Q8TEM1;P57740;O75694;P35658;Q8WUM0;P52948-5
Rev-mediated nuclear export of HIV RNA	9	40	0.005956059	P52948-3;P52948-4;Q92621;Q8TEM1;P57740;O75694;P35658;Q8WUM0;P52948-5
NEP/NS2 Interacts with the Cellular Export Machinery	9	42	0.008041143	P52948-3;P52948-4;Q92621;Q8TEM1;P57740;O75694;P35658;Q8WUM0;P52948-5
Interactions of Rev with host cellular proteins	9	42	0.008041143	P52948-3;P52948-4;Q92621;Q8TEM1;P57740;O75694;P35658;Q8WUM0;P52948-5
Export of Viral Ribonucleoproteins from Nucleus	9	43	0.009271901	P52948-3;P52948-4;Q92621;Q8TEM1;P57740;O75694;P35658;Q8WUM0;P52948-5
Vpr-mediated nuclear import of PICs	9	44	0.010639957	P52948-3;P52948-4;Q92621;Q8TEM1;P57740;O75694;P35658;Q8WUM0;P52948-5
Interactions of Vpr with host cellular proteins	9	47	0.015656599	P52948-3;P52948-4;Q92621;Q8TEM1;P57740;O75694;P35658;Q8WUM0;P52948-5
SUMOylation of RNA binding proteins	9	51	0.024796641	P52948-3;P52948-4;Q92621;Q8TEM1;P57740;O75694;P35658;Q8WUM0;P52948-5
Interleukin-12 family signaling	9	56	0.040866683	Q13126;P42224;P37837;P14174;P61224;Q53EL6;P13796;P04179;P40763
ROS and RNS production in phagocytes	8	36	0.009890643	Q15080;P19878;P15153;P21283;P21281;P36543;P04839;P38606
tRNA Aminoacylation	8	42	0.02254219	P54577;Q9NSD9;Q9H2U2;P26639;Q15181;Q12904;O43776;Q9NSE4
TBC/RABGAPs	8	45	0.031942694	P62330;P61020;Q9NVG8;P62491;P61006;Q9UJY4;P20340;P51149
Golgi Cisternae Pericentriolar Stack Reorganization	7	14	1.73E-04	Q9H0U4;O60763;P28482;P27361-3;Q9H8Y8;P61019;P62820
GRB2:SOS provides linkage to MAPK signaling for Integrins	7	15	2.61E-04	P05106;P08514;P62993-1;P61224;P12931;Q7Z5R6;P02751
Postmitotic nuclear pore complex (NPC) reformation	7	28	0.00849116	Q8WYP5;Q92621;Q92973;P57740;O75694;Q8WUM0;P52948-5
The role of Nef in HIV-1 replication and disease pathogenesis	7	29	0.010145797	Q92608;P63000;Q10567;Q96CW1;O95782;Q92556;O43747
GPVI-mediated activation cascade	7	35	0.02528163	P84095;P43405;P15153;P63000;P29350;P61586;P16885
RHOH GTPase cycle	7	37	0.032639474	P52565;P41240;P11182;O75323;P52566;P51149;P27105
Gene and protein expression by JAK-STAT signaling after Inter	7	37	0.032639474	Q13126;P37837;P14174;P61224;Q53EL6;P13796;P04179
p130Cas linkage to MAPK signaling for integrins	6	15	0.001555115	P05106;P08514;P61224;P12931;Q7Z5R6;P02751
Signal transduction by L1	6	21	0.007840027	P05106;P08514;P28482;P63000;P27361;Q02750
Mitochondrial calcium ion transport	6	23	0.011850097	O75439;O95202;Q9Y4W6;Q10713;P21796;Q8NE86
Cytosolic tRNA aminoacylation	6	24	0.014317974	P54577;Q9NSD9;P26639;Q15181;Q12904;O43776
Signaling by CSF3 (G-CSF)	6	30	0.036821516	P42224;Q15369;P43405;P62993;Q15370;P40763
Signaling by CSF1 (M-CSF) in myeloid cells	6	31	0.041996401	P42224;Q92835;P62993-1;P12931;P40763;P16885
Glyoxylate metabolism and glycine degradation	6	31	0.041996401	P30038;P36957;P08559;P11182;Q9UBQ7;P11177
Tat-mediated HIV elongation arrest and recovery	6	32	0.047607015	Q15369;Q15370;Q08945;P19387;O00267;Q9Y5B9
Pausing and recovery of Tat-mediated HIV elongation	6	32	0.047607015	Q15369;Q15370;Q08945;P19387;O00267;Q9Y5B9
mitochondrial fatty acid beta-oxidation of saturated fatty acic	5	11	0.002199413	Q16836;Q9BV79;P30084;P55084;P49748
Advanced glycosylation endproduct receptor signaling	5	13	0.004449658	P17931;P28482;P27361;P04271;P39656
Spry regulation of FGF signaling	5	16	0.010341717	P62993-1;P12931-1;P28482;P27361;P62714
Nuclear events stimulated by ALK signaling in cancer	5	20	0.024392663	P28482;P27361;Q9HC35;P02751;P40763
VxPx cargo-targeting to cilium	5	21	0.029201732	Q92538;P62491;P18085;Q96A65;P61006
Signaling by phosphorylated juxtamembrane, extracellular an	5	22	0.034570035	P42224;P62993-1;P12931-1;P42224-1;P40763
ERK/MAPK targets	5	22	0.034570035	P51452;Q15418;P28482;P27361;P62714
Signaling by KIT in disease	5	22	0.034570035	P42224;P62993-1;P12931-1;P42224-1;P40763
Growth hormone receptor signaling	5	24	0.047033681	P42224;P28482;P27361;P29350;P40763
Beta oxidation of decanoyl-CoA to octanoyl-CoA-CoA	4	6	0.001555045	Q16836;Q9BV79;P30084;P55084
TYSND1 cleaves peroxisomal proteins	4	7	0.002704541	P22307;P51659;O00116;Q15067
NFE2L2 regulates pentose phosphate pathway genes	4	8	0.004332168	P29401;P37837;P52209;P11413
Cohesin Loading onto Chromatin	4	10	0.009329946	Q9UQE7;Q9NTI5;Q8N3U4;Q29RF7
Establishment of Sister Chromatid Cohesion	4	11	0.012833408	Q9UQE7;Q9NTI5;Q8N3U4;Q29RF7
ALK mutants bind TKIs	4	12	0.017079432	Q9HC35;P10644;O43815;P02751
PECAM1 interactions	4	12	0.017079432	P05106;Q92835;P12931-1;P29350
ERKs are inactivated	4	13	0.022109819	P51452;P28482;P27361;P62714
Mitotic Telophase/Cytokinesis	4	13	0.022109819	Q9UQE7;Q9NTI5;Q8N3U4;Q29RF7
alpha-linolenic acid (ALA) metabolism	4	13	0.022109819	P22307;P51659;P33897;Q15067-2
alpha-linolenic (omega3) and linoleic (omega6) acid metabolis	4	13	0.022109819	P22307;P51659;P33897;Q15067-2
Pentose phosphate pathway	4	15	0.034639099	P29401;P37837;P52209;P11413
Glutamate and glutamine metabolism	4	15	0.034639099	P04181;O94925;Q96C36;P32322
Processing of SMDT1	4	16	0.042170644	O75439;Q9Y4W6;Q10713;Q8NE86
Signaling by MAP2K mutants	3	4	0.004404576	P28482;P27361;Q02750
Beta oxidation of hexanoyl-CoA to butanoyl-CoA	3	5	0.008107751	Q16836;P30084;P55084
Beta oxidation of octanoyl-CoA to hexanoyl-CoA	3	5	0.008107751	Q16836;P30084;P55084
Beta oxidation of lauroyl-CoA to decanoyl-CoA-CoA	3	5	0.008107751	Q16836;P30084;P55084
Negative feedback regulation of MAPK pathway	3	6	0.013207524	P28482;P27361;Q02750
RSK activation	3	7	0.019776592	Q15418;P28482;P27361
Fructose metabolism	3	7	0.019776592	Q00796;P15121;Q3LXA3
Sema4D mediated inhibition of cell attachment and migratior	3	8	0.02784395	P63000;P10301;P61586
Cross-presentation of particulate exogenous antigens (phago:	3	8	0.02784395	Q15080;P19878;P04839
Lipophagy	3	9	0.037403073	Q9Y478;Q99541;P54619
Nef and signal transduction	3	9	0.037403073	Q92608;P63000;Q92556
Signal attenuation	3	10	0.048418951	P62993-1;P28482;P27361
Insulin effects increased synthesis of Xylulose-5-Phosphate	2	2	0.011706049	P29401;P37837
Fructose biosynthesis	2	2	0.011706049	Q00796;P15121
Beta oxidation of palmitoyl-CoA to myristoyl-CoA	2	3	0.024983824	P55084;P49748
Pyrophosphate hydrolysis	2	3	0.024983824	Q9H2U2;Q15181
Axonal growth stimulation	2	4	0.042146602	P52565;P61586
Biosynthesis of protectins	2	4	0.042146602	P09960;P16050
Galactose catabolism	2	4	0.042146602	P51570;Q14376
Biosynthesis of E-series 18(R)-resolvins	2	4	0.042146602	P09960;P16050
