# Supplementary material for: Proximity-dependent mapping of the HCMV US28 interactome identifies RhoGEF signaling as a requirement for efficient viral reactivation
Source: PLoS Pathog. 2023 Oct 2;19(10):e1011682. doi: 10.1371/journal.ppat.1011682 (PMC10569644; doi:10.1371/journal.ppat.1011682)
Supplement: Attachment — Submitted filename: Rebuttal for US28 Interactome Paper.docx [file ppat.1011682.s007.docx]

**RE: Proximity-Dependent Mapping of the HCMV US28 Interactome Identifies RhoGEF Signaling as a Requirement for Efficient Viral Reactivation**

First, we would like to thank the reviewers for their careful evaluation of our manuscript. We were happy that the reviewers found the study interesting. Based upon the reviewers comments we have made substantial changes to the manuscript including additional experiments to show that US28 trafficking is not grossly affected by the BirA Turbo fusion; re-ran transfection reporter assays to include the Renilla control plasmid; demonstrate anti-RhoA activity for Rhosin and Y16; and most substantially, we have performed a US28 BirA Turbo proximity labeling experiment in HCMV latently infected CD34+ HPCs that supported the finding of the RhoA pathway as part of the HCMV US28 interactome. Comments to the specific reviewer concerns are listed below:

**Reviewer #1**

1. Fig. 1: The authors present data demonstrating that the addition of TurboID does not appear to negatively impact US28 signaling. However, it is important to also demonstrate that the addition of TurboID does not alter US28 localization. This is a critical control given that localization could significantly impact the US28 interactome.

*We agree with the reviewer that the TurboID tag could impact localization of US28. Therefore, we have performed an immunofluorescence microscopy experiment to track US28 expression in transfected human fibroblasts expressing either pcDNA3.1-HA-US28 or pcDNA3.1-HA-US28-TurboID. We did not observe any discernable differences in the localization of US28-TurboID relative to US28 alone (new Figure 1D).*

2. Given that HEK293M cells are not permissive for HCMV replication, the authors should provide a justification as to why these cells were chosen for this study.

*HEK293M cells were used initially for exploring the interactome using transfection conditions to validate that the tag did not interfere with signaling prior to construction of the TB40/E-GFP-US28-TurboID constructs. We and others have observed that US28 signaling in 293 cells is a robust system for initial interrogation of signaling pathway inhibitors. While 293 cells were used for initial characterization, the interactome and inhibitors were shown to have similar effects when looking at virally infected cells. We have added further justification for using 293 cells in the results section of the paper. In addition, we now present US28 interactome data for latently infected CD34+ HPCs that corroborates the linkage with the RhoA pathway.*

3. Fig. 3a: US28 interactions were validated from HEK293M cell transfected by a HA-tagged US28-TurboID protein (US28-BT-HA) construct. However, these interactions should be validated in the context of a viral infection in NHDF (or another permissive cell line) as the authors have already generated a HCMV TB40E-GFP-TurboID (BT).

*We agree with the reviewer that identified proteins should be validated within the context of infection. We have since repeated these experiments with human fibroblasts infected with TB40/E-GFP and TB40/E-GFP-US28-TurboID as illustrated in Figure 5A (previously Figure 4).*

4. Fig. 3B lacks statistics. The labelling is also unclear. Presumably ARCHGEFs represent siRNAs against different RhoGEFs? TIRO needs to be defined in the figure legend. There is no scrambled control siRNA treatment group. The authors provide no evidence that the siRNA worked or how efficient the knockdown was.

In this figure the mock treated samples were performed using a scrambled control siRNA; we apologize for not stating that clearly. *The siRNAs used in our experiments were purchased from ThermoFisher and the siRNA pools are guaranteed to target specific RhoGEFs. As stated above the dearth of specific antibodies against RhoGEFs has been challenging to show knockdown but the data compliments our inhibitor data using an orthogonal assay.*

5. Fig 3C: The authors use 2 Rho inhibitors but provide no evidence that either inhibitor is working as expected in their model system.

*We thank the review for this thoughtful comment; we have since explored the efficacy of Rhosin and Y16 ability to inhibit activation of RhoA in a human fibroblast cell line as shown in Supplemental Figure 1*. R*hosin and Y16 work as expected to decrease RhoA activation in a dose-dependent manner when compared to untreated cells.*

6. Supplementary data appears to be mislabeled and/or missing. Cytotoxicity studies (Fig. S2A) could not be found.

*We thank the reviewer for this comment and have added the missing information to Fig. S1A (Previously Fig. S2A).*

7. Fig. 4. Although the authors validate US28 interactors with transient transfections in HEK293M cells, US28 interaction with RhoGEFs needs be validated in the context of latency. This may be difficult with CD34+ cells, however THP-1 cells, which allow for HCMV latency and the expression of US28 during latent infection, could be used.

*We agree with the reviewer that these phenomena need to be validated within the context of latent infection. Therefore, we performed a US28-TurboID proximity labeling experiment in CD34^+^ HPCs and found that the RhoA pathway including RhoGEFs were part of the US28 interactome, which corroborates our data in 293 and NHDFs. This new data can be found in Figures 3E/F, 4A/B and Table 2. The CD34+ HPC interactome adds an important new level of understanding for both host and viral proteins that are in close proximity to US28 during infection.*

8. Fig. 5: The authors provide evidence that treatment with Rhosin prevents HCMV reactivation in vivo based on HCMV copy number following G-CSF treatment. Is it not possible that Rhosin simply attenuates HCMV replication, which leads to lower HCMV copy numbers? The authors state in Fig.S2B (which could not be found), Rhosin had no effect on HCMV replication in vitro. However, this may not be the case in vivo. Mice could be treated with Rhosin following G-CSF reactivation to demonstrate that Rhosin has no effect of viral replication rates in vivo.

*We thank the reviewer for this comment, and we feel that our observations suggest that the effect of Rhosin is altering the transitional events that lead to reactivation from latency and does not effect lytic replication. We do not observe a reduction in HCMV replication in fibroblasts treated with either compound. We have since added the missing figure showing that treatment with Rhosin or Y16 does not have any effect on HCMV replication in vitro (Fig. S1B).*

9. Overall, the authors conclude that blocking US28-RhoGEF interactions prevents HCMV reactivation. Although it is clear that RhoGEF signaling is required, the authors do not provide direct evidence that blocking US28-RhoGEF interactions prevents viral reactivation. At least showing that Rhosin (a steric inhibitor) blocks US28 interaction with RhoGEFs would provide support that the interaction is necessary for HCMV reactivation.

*In GPCR signaling complexes, activation of the receptor releases the Gα subunit that mediates direct activation of the RhoGEF. The active form of the RhoGEF then interacts with RhoA providing GDP-GTP exchange and activation of RhoA. We do not believe that Rhosin blocks US28:RhoGEF interactions but rather block specific RhoGEF interactions with RhoA.*

**Reviewer #2**

1. For the reporter assays in Figure 1C and 1D, were internal renilla luciferase control levels measured and used to correct the SRE and SRF reporter data? Could the data be recalculated and shown as a fold over the basal (empty vector)? As shown it is difficult to ascertain the level of US28 based signaling compared to the empty vector?

*We agree with the reviewer and have performed the signaling assays again and have included the renilla-luciferase as a control. The corrected signaling data for SRE and SRF-RE as shown in Figure 1B and 1C.*

2. A more granular and easy to follow analysis of the comparison between transfected and infected cell US28 interactome would be useful. While Figure 2 attempts to do this, it is not sufficient. As an example, in the TB40E interactome in 2C, Rho GTPase Cycle is listed twice with different number of proteins, while 2A and 2C differ with 2A listing RhoA GTPase effectors. Both list “Signaling by Rho GTPases” but it is not clear if these represent the same or significantly overlapping proteins.

*The pathways listed in Figure 3 were derived from the Reactome pathway analysis tool, which does contain similar and sometimes overlapping pathways. We have amended the figure to try to reduce the overlap as suggested by the reviewer. We also provide a Venn diagram in Figure 4 that highlights some of the differences and similarities between the viral and host proteins identified in proximity with US28 in NHDF vs. CD34^+^ HPCs.*

3. While the pulldowns in Figure 3A look clean and interacting proteins are absent in the non-BirA expressing cells, an additional control showing these pull downs in cells expressing an irrelevant BirA expression vector would be more convincing. The authors only look at p115-RhoGEF, have the authors done similar experiments with other Rho-GEFs.

*To get at this question, we repeated the pulldown experiments for US28 interactome proteins in HCMV infected NHDFs. While we are limited by the available reagents to RhoGEFs, we now provide data for two independent RhoGEFs (PDZ-RhoGEF-ARHGEF11 and p115-RhoGEF ARHGEF1). Both were present in the interactome and validated by pulldown assays.*

4. Mechanistic information regarding how interaction between US28 and the Rho-GEFs leads to activation of Rho is lacking. Does US28 cause a relocalization of Rho-GEF that facilitates GTP loading onto Rho? Does US28 cause an increase in GTP bound Rho? Can the requirement for US28 in reactivation become overcome by indirectly activating Rho or by expressing constitutively active mutants of Rho?

*We thank the reviewer for their comment and have since added further clarification to the manuscript. Our current idea about how US28 activates RhoGEFs is through G-protein activation following the normal signal transduction pathway. G proteins can bind and directly activate RhoGEFs and we’ve shown previously that US28 signaling through Gα_12/13_ can mediate RhoA activation (PMC446127).*

5. In Figure 3B, it is not clear which RhoGEF is targeted by the indicated siRNAs. Are they all p115-RhoGEF specific? Moreover, there are no error bars and it is not clear as to how many times this experiment was repeated. In Figure 3C, the effects of Y16 and Rhosin seem significant. However, it is again unclear if an internal constitutive renilla reported was used to control for the firefly luciferase and if the pharmacological inhibitors could have in fact blocked both the reporter and the constitutive renilla construct.

*The siRNAs used in our experiments were designed to target specific RhoGEFs according to the ThermoFisher website where they were purchased from; and the pools that we purchased are guaranteed to work. As stated above the dearth of specific antibodies against RhoGEFs has been challenging. The pharmacological inhibitors experiment in Figure 5C was redone to include the renilla reporter that was used to normalize each sample.*

6. In Figure 4A the results of one experiment are shown, while in Figure 4B the combined results of 4 independent donors are shown. In Figure 4B the results are presented as fold change relative to the reactivation control, which in each experiment is set to 1.0. This eliminates the variability of the reactivation control and I am not certain that the statistics are vaild. I can appreciate that the frequency of infectious centers would be variable from donor to donor, which is why the data is set to 1.0 for the reactivation control. Perhaps the data in Figure 4B can be superimposed with the individual data points from the 4 experiments or the actual average frequency of infectious centers could be displayed for the 4 independent experiments.

*We thank the reviewer for their comment and have since added the representative replicates within Supplemental Figure 3. While only the relative frequency of infectious centers are shown, the general trend within the data remains constant and we believe the validity of our statical analysis. Figure 6 now contains the merged data for the four donors and we have put the graphs for each individual into Supplmental information (Fig. S2). We hope that this will simplify the figure without detracting or losing information.*

7. The experiments in Figure 4 should be shown with both wildtype and US28 null viruses. Since the authors are claiming that US28 signaling through Rho is important for reactivation it would be important to show the levels of reactivation for US28 null virus as well as the effects of the inhibitors on reactivation of US28 null virus. Moreover, in vivo experiments in Figure 5 assessing the effects of Rhosin on inhibiting reactivation should similarly be performed with both wildtype and US28 null viruses.

*The point is well taken; however, we previously reported that US28-null virus is incapable of reactivating both in vitro and in vivo. We observe a similar phenotype with Rhosin treatment in vitro and in vivo. Thus, we would not expect to see any difference (i.e., enhancement of reactivation nor suppression since it is already lacking for the mutant virus).*

**Reviewer #3**

1. A major concern is the overinterpretation of the BioID data. As properly stated in lines 50, 98, 163 and 488, this assay detects proximal proteins. As shown in Figure 1A, this could include interacting proteins or those simply nearby. The terms interaction(s), interactome and interactor are used heavily throughout the manuscript (40, 41, 55, 96, 110, 112,114, 127, 148, 159, 161, 170, table 1, etc.), and even in the title. However, no work has been done to confirm whether any of these hits are actually interacting with US28, as opposed to just in proximity to US28. In fact, the statement on line 267 “We confirmed the physical interaction between US28 and several proteins from our proteomic analysis” is actually false. Given that the proteins were pulled down with NeutrAvidin beads, this experiment confirmed only that they were biotinylated, meaning in close proximity to US28, not that they were physically interacting. These hits should be investigated for a physical interaction using co-immunoprecipitations (or other appropriate assay).

*We thank the reviewer for their comment and agree that biotinylation does not necessarily constitute a direct interaction. We have since changed the wording within the manuscript for clarification.*

2. Given the differences observed between transfection and infection, particularly with the STRING analysis, the relevancy of the transfection data is questionable with respect to making any conclusions about infection. The results in figure 3B would therefore be better in the context of infection, similar to how the results of 3C were shown for infection in Figure 4. Even if signaling is decreased following knockdown of the targets after transfection, this may or may not be applicable in the context of the virus. An additional concern with Figure 3B is the lack of any confirmation that the respective targets were knocked down. This is an essential piece of data for interpreting the results and a western blot should be included.

*As mentioned above the lack of specific antibodies directed against RhoGEFs has limited our ability to demonstrate knockdown. However, the siRNAs used in our experiments were designed to target specific RhoGEFs. Importantly, the siRNA approach was used to determine whether the Rho pathway was important for US28 signaling and to provide an orthogonal approach to the pharmacological inhibitors used to target this pathway.*

3. The use of the humanized mouse model to show that inhibition of Rho-GEFs via Rhosin prevents reactivation of latency is a strength of this paper. However, the conclusions of this experiment are overstated. Line 227-228 states that “This data demonstrates that US28-RhoGEF interactions contribute to viral reactivation.” The results do show a role for RhoGEFs in reactivation, which is inhibited by Rhosin, but the link to US28 is assumed. Other pathways involving RhoGEFs could equally be involved. No conclusions can be made about US28 from the data as currently presented. This conclusion requires breaking the US28-RhoGEF link, not just inhibiting the Rho-GEFs. One way to show this could involve identifying mutations on US28 that break that link.

*We agree with the reviewer and have modified our comments to reflect the importance of RhoGEF in viral reactivation.*

**Reviewer #1**

There are some minor typos throughout the manuscript.

*We thank the reviewer for bringing this to our attention and have carefully gone through the manuscript to fix any outstanding typos.*

**Reviewer #2**

1. Supplemental Figure 2A appears to show the data as a log scale, but it the text the authors refer to drug concentrations up to 40uM. This could be shown more clearly. Moreover, although the drugs are not toxic to fibroblasts, the authors have not assessed toxicity in HPCs, which would be more relevant to the current study, which analyzes the effects of these drugs on HCMV reactivation in HPCs.

*We thank the reviewers for their comment and have amended Supplemental Figure 1 to account for the log transformation. Additionally, we have conducted cytotoxicity experiments in CD34^+^ HPCs which confirmed that neither compound is toxic at relevant concentrations.*

2. The RT-qPCR data should also indicate the more common Rho-GEF nomenclature (p115-RhoGEF, PDZ-RhoGEF, etc.) in addition to the actual gene name.

*We appreciate the comment and have added the common nomenclature.*

**Reviewer #3**

1. The source of the cells, either 293M or NHDF, are not included in the Materials and Methods

*The cells were originally obtained from Microbix and ATCC, this information has been added to the Materials and Methods.*

2. For Supplemental Figure 3, there is a clear difference in transcript levels between cells as can be deduced from Ct values. However, a standard curve would allow for quantification and provide actual numbers for the differences in transcript levels between the two cell types.

*Using the ΔΔCt method we compared relative levels of RhoGEFs to a control transcript and the assay was performed with predesigned, commercially available primers and probes. The analysis was merely used to show that the RhoGEFs targeted by Rhosin and Y16 are transcribed in the cell types used in our study. A relative standard curve would ideally show exactly the same outcome, that the RhoGEFs are indeed transcribed in these cell types.*

3. Figure 4A shows the data for one HPC donor. It would be nice to include in Figure 4A the data from all four donors that was used to calculate data for Figure 4B to allow for a sense of variability amongst donors.

*We thank the reviewer for their comment and have added the representative replicates within Supplemental Figure 3. While only relative frequency of infectious centers are shown, the general trend within the data remains constant and we believe the validity of our statistical analysis.*

4. Figure 6 contains inactive and active versions of US28, but there is no discussion of the different US28 versions at all in the text. Searching for the term inactive returns no hits. Please provide an explanation in the text or adjust the figure appropriately.

*We have modified this figure. Originally the words were meant to reveal that ligand binding to US28 activates the receptor not that we used inactive or active versions of the receptor.*

5. Line 234 states that “Our findings are consistent with a recently published US28 proximity labeling study”, yet many of the top hits between the two studies do not appear to overlap. Was EphA2 found in your study? That study identified only ARHGEF17, but not other RhoGEFs. There should be more discussion on the similarities and the differences between the two studies, and the claim on line 234 is a bit misleading.

*We have added more discussion around the similarities to our results and those from others and compared the interactome obtained from NHDFs vs. CD34+ HPCs. Interestingly, we picked up EphA2 in US28 proximity labeling in infected NHDFs but not in latently infected CD34+ HPCs indicating a cell-type specificity to the US28 interactome. However, the Rho pathway (including RhoGEFs) was identified in both interactomes suggesting that this pathway is intimately linked to US28 signaling regardless of cell type.*
