# Supplementary material for: Proximity-dependent mapping of the HCMV US28 interactome identifies RhoGEF signaling as a requirement for efficient viral reactivation
Source: PLoS Pathog. 2023 Oct 2;19(10):e1011682. doi: 10.1371/journal.ppat.1011682 (PMC10569644; doi:10.1371/journal.ppat.1011682)
Supplement: Attachment — Submitted filename: Rebuttal for US28 Interactome Paper_8.14.2023.docx [file ppat.1011682.s008.docx]

**RE: Proximity-Dependent Mapping of the HCMV US28 Interactome Identifies RhoGEF Signaling as a Requirement for Efficient Viral Reactivation**

First, we would like to thank the reviewers for their careful evaluation of our revised manuscript. We have amended the current manuscript to reflect the additional comments that we received. Below is a rebuttal to the reviewers’ comments:

**Part III – Minor Issues: Editorial and Data Presentation Modifications**

Reviewer #1:

1. Although the western blots are clear and support their conclusions, the figure legends should include the number of biological replicates that were performed for each immunoblot.

*We thank the reviewers for their suggestion and have added the requested information to the appropriate figure legends.*

Reviewer #2

1. In Figures 2C and 2D, the Streptavidin-HRP blots are from whole cell extracts (unbound), not pull downs, correct? It is curious that there are far more proteins labelled in the infected NHDFs and CD34^+^ cells than in the HEK cells. The authors should comment on this or clarify.

*We have amended the figure, legend, and manuscript text to indicate the correct sample source for each experiment. To provide clarity regarding the difference in protein abundances between transfected HEK293 cells and infected CD34^+^ HPCs and NHDFs, the streptavidin HRP blots from the HEK293s are post bead-based purification whereas the streptavidin HRP blots from NHDFs/CD34+ HPCs are whole cell lysates.*

2. It is interesting that US28 shows up in the list of viral interacting proteins in NHDFs, but not CD34^+^ cells. One would think that the BirA would biotinylate the US28 that it is fused with and then show up in both NHDFs and CD34+ cells. The authors should comment on this or clarify.

*During latent infection, viral proteins are sparingly expressed. Therefore, it is not too surprising that we only identified US28 in the NHDF interactome data set, but this finding would again support the notion that even very little US28 can have a dramatic effect on the latently infected cell and it’s ability to reactivate.*

3. In Figure 5B, the SRF reporter assay data does not show statistical significance or error bars. In the text of the manuscript these data are interpreted as a “marked decrease of US28 signaling”. Moreover, from the figure legend it is not apparent if these data were corrected with renilla luciferase as an internal control?

*We have removed this data from the figures, legends, and, manuscript text.*

4. Supplemental Figure 1 labelling is inconsistent between the text, legend, and figure. This needs to be corrected.

*We have since amended the figure, legend, and manuscript text to address any inconsistencies.*

5. The authors should carefully review all figure panels to ensure that all legends and panels match the text.

*We have since amended the figure, legend, and manuscript text to address any inconsistencies.*

6. Since Figure 6 is an average of the 4 independent donors (which are shown in Figure S3), this should be mentioned in the text in the results section. If possible, the individual data points from Figure S3 could be overlayed onto the data in Figure 6, which would help to better represent the variability between individual donors/experiments.

*We have amended the figure legend and manuscript text to indicate that results are presented as an average of four independent experiments. Additionally, we have overlayed individual data points from Figure S3 into Figure 6 to make this more apparent.*
